# The Temporal Expression of Global Regulator Protein CsrA Is Dually Regulated by ClpP During the Biphasic Life Cycle of *Legionella pneumophila*

**DOI:** 10.3389/fmicb.2019.02495

**Published:** 2019-11-07

**Authors:** Zhen-huang Ge, Qin-sha Long, Pei-bo Yuan, Xin Pan, Dong Shen, Yong-jun Lu

**Affiliations:** ^1^School of Life Sciences, Sun Yat-sen University, Guangzhou, China; ^2^Biomedical Center, Sun Yat-sen University, Guangzhou, China

**Keywords:** *Legionella pneumophila*, biphasic life cycle, CsrA, ClpP, IHFB

## Abstract

*Legionella pneumophila*, an environmental bacterium that parasitizes protozoa, is the causative pathogen of Legionnaires' disease. *L. pneumophila* adopts a distinct biphasic life cycle that allows it to adapt to environmental conditions for survival, replication, and transmission. This cycle consists of a non-virulent replicative phase (RP) and a virulent transmissive phase (TP). Timely and fine-tuned expression of growth and virulence factors in a life cycle-dependent manner is crucial. Herein, we report evidence that CsrA, a key regulator of the switch between the RP and the TP, is dually regulated in a ClpP-dependent manner during the biphasic life cycle of *L. pneumophila*. First, we show that the protein level of CsrA is temporal during the life cycle and is degraded by ClpP during the TP. The ectopic expression of CsrA in a Δ*clpP* mutant, but not in the wild type, inhibits both the initiation of the RP *in vitro* and the invasiveness to *Acanthamoeba castellanii*, indicating that the ClpP-mediated proteolytic pathway regulates the CsrA protein level. We further show that the temporally expressed IHFB is the transcriptional inhibitor of *csrA* and is degraded *via* a ClpP-dependent manner during the RP. During the RP, the level of CsrA is increased by promoting the degradation of IHFB and reducing the degradation of the accumulated CsrA *via* a ClpP-dependent manner. During the TP, the level of CsrA is decreased by inhibiting the degradation of IHFB and promoting the degradation of the accumulated CsrA *via* a ClpP-dependent manner as well. In conclusion, our results show that the growth-stage-specific expression level of CsrA is dually regulated by ClpP-dependent proteolysis at both the transcription and protein levels during the biphasic life cycle of *L. pneumophila*.

## Introduction

*Legionella pneumophila* is a Gram-negative intracellular bacterial pathogen that is the causative agent for most cases of Legionnaires' disease (Fields et al., 2002; Newton et al., 2010; Guyard and Low, 2011). *L. pneumophila* has a biphasic life cycle that allows it to benefit from the environment of the susceptible host cell and simultaneously ensure its persistence for another infection cycle (Oliva et al., 2018). Within host cells, the bacteria differentiate into two forms—replicative and transmissive—undergoing physiological, morphogenetic, and metabolic changes (Molofsky and Swanson, 2004; Bruggemann et al., 2006). In broth culture, the bacteria enter exponential and post-exponential forms, requiring similar physiological, morphogenetic, and metabolic changes (Byrne and Swanson, 1998; Hammer and Swanson, 1999). The gene expression programs in replicative and transmissive bacteria *in vivo* are similar to that of exponential and post-exponential bacteria *in vitro*, respectively, suggesting that the biphasic life cycle is controlled globally by the bacterial growth phase and/or nutrient availability (Oliva et al., 2018). The transition from exponential/replicative phase (RP) to post-exponential/transmissive phase (TP) is governed by a common virulence program (Bruggemann et al., 2006; Faucher et al., 2011). Therefore, the exponential and post-exponential phase in broth cultures has been used to emulate the RP and TP of the *L. pneumophila* life cycle (Bruggemann et al., 2006).

The biphasic life cycle of *L. pneumophila* is crucial for the fitness of the pathogen and is linked to its metabolism (Molofsky and Swanson, 2004). *L. pneumophila* employs at least four distinct two-component systems (TCSs), including LetA/S, PmrA/B, LsqR/ST, and CpxR/A, which govern its differentiation from the replicative to the transmissive state (Gal-Mor and Segal, 2003; Jacobi et al., 2004; Tiaden et al., 2007; Zusman et al., 2007; Altman and Segal, 2008). However, the underlying regulatory cascades and environmental cues controlling this dimorphism are poorly understood. A key regulator of the switch between RP and TP in *L. pneumophila* is the carbon storage regulator CsrA, a pivotal repressor of transmission traits and activator of replication (Molofsky and Swanson, 2003; Forsbach-Birk et al., 2004). CsrA is a posttranscriptional regulator that represses a variety of post-exponential phase genes in bacteria, which plays important roles in regulating motility, virulence, and metabolism (Vakulskas et al., 2015). CsrA was reported to bind more than 450 mRNA targets in *L. pneumophila*, altering their translation, transcription, and/or stability (Molofsky and Swanson, 2003; Sahr et al., 2009, 2017). This indicates that CsrA is indispensable and plays an essential role in the life cycle (Molofsky and Swanson, 2003; Sahr et al., 2017). Three TCSs—LetS/LetA, PmrB/PmrA, and LqsS/LqsT/LqsR—have been shown to regulate CsrA activity (Vakulskas et al., 2015). However, the regulatory factors that directly fine-tune the timing of CsrA expression have not yet been identified in *L. pneumophila* or any other bacterium.

Regulation of gene expression by controlled proteolysis contributes to the survival of pathogenic bacteria during host interaction. This mechanism was first elucidated by the discovery of several global regulatory proteins that are under proteolytic control (Gottesman, 2003; Mahmoud and Chien, 2018). ClpP, the catalytic core of the Clp proteolytic complex, is highly conserved in bacteria and widely involved in many cellular processes by regulating intracellular protein quality (Mahmoud and Chien, 2018). Indeed, ClpP is required for the intracellular proliferation of *L. pneumophila* in both amoeba and murine macrophages (Li et al., 2010; Zhao et al., 2016) as well as for optimal translocation of several effector proteins (Zhao et al., 2016). ClpP also plays an important role in cell division and the expression of transmission traits of *L. pneumophila*, suggesting a putative role for ClpP in the regulation of its life cycle (Li et al., 2010). However, the underlying regulatory mechanism affected by ClpP in transition between the RP and the TP is poorly understood.

In this study, we investigated how CsrA expression during the life cycle of *L. pneumophila* is regulated in a finely tuned and temporal manner that is dependent on ClpP. We found that CsrA is required for bacterial cells in the TP to passage into the RP. We further demonstrated that ClpP regulates the expression of CsrA, thereby controlling the inhibitory function of CsrA during intracellular proliferation. Finally, we showed that IHFB is a transcriptional inhibitor of CsrA. During the RP, the level of IHFB decreases, allowing transcription and elevated protein levels of CsrA. This pairs with a decrease in CsrA clearance, with both effects dependent on ClpP. In contrast, during the TP, ClpP proteolysis degrades accumulated CsrA and allows IHFB to increase, cutting off transcription of CsrA. These findings reveal the temporal regulation mechanism of CsrA by ClpP during the biphasic life cycle of *L. pneumophila*.

## Results

### CsrA Is Temporally Expressed During the Life Cycle and Is Regulated by ClpP

**Figure 1:**
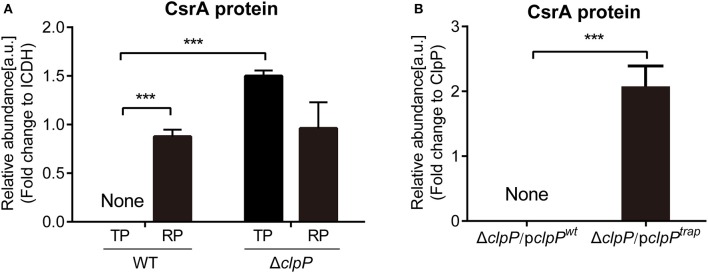
Proteomics analysis of CsrA and identification of CsrA as a substrate of ClpP. **(A)** The abundance of CsrA in WT and Δ*clpP* at indicated growth phases. WT and Δ*clpP* were inoculated into fresh AYE medium at the same initial OD_600_ values. Bacterial cells in the RP were harvested at an OD_600_ of 0.7–1.0 and those in the TP were harvested approximately 6 h after the cessation of growth. Total proteins from indicated samples were extracted for proteomic analysis, and the representative peptides were identified by mass spectrometry. ICDH was measured as a loading control. Data represent mean ± SD derived from three independent experiments. ***p < 0.001 were identified by GraphPad Prism. **(B)** The abundance of CsrA in Δ*clpP*/p*clpP*^*wt*^ and Δ*clpP*/p*clpP*^*trap*^ in the TP. Bacterial cells were harvested approximately 6 h after the cessation of growth. Bacterial whole-cell lysates from Δ*clpP*/p*clpP*^*wt*^ and Δ*clpP*/p*clpP*^*trap*^ were prepared and His-tagged proteins were purified by Ni-NTA affinity. Substrates captured inside the proteolytic barrel were co-purified along with the His-tagged ClpP complex and identified by mass spectrometry. ClpP was measured as a loading control. Data represent mean ± SD derived from three independent experiments. ***p < 0.001 were identified by GraphPad Prism.

In order to assess the protein level of CsrA at different growth phases, we performed proteomic analysis of whole lysates obtained from cultures of *L. pneumophila* wild-type (WT) strain grown in liquid medium at indicated time points. The results showed that CsrA was not detected during the TP, while high levels of CsrA were detected upon entry into the RP (Figure 1A). Thus, the presence of CsrA during the biphasic life cycle of *L. pneumophila* is growth-phase-dependent.

Our previous work suggested a putative role for the protease ClpP in the regulation of life cycle and virulence in *L. pneumophila* based on the behavior of a *clpP* deletion mutant, Δ*clpP* (Li et al., 2010; Zhao et al., 2016). We designed experiments to determine whether the regulation of CsrA is associated with ClpP. To validate the mutant strain, the whole genomes of WT and Δ*clpP* were re-sequenced and compared (WT Accession: LP02, PRJNA522676; Δ*clpP*Accession:XP02, PRJNA522681). The results showed that only the *clpP* gene had been specifically knocked out (Supplementary Figure S1). Therefore, the *clpP* allele complemented strain will not be included in the experiments below where indicated.

The protein levels of CsrA in Δ*clpP* were determined by proteomic analysis of whole lysates of *L. pneumophila* grown in liquid medium and collected during different growth phases (Supplementary Figure S2). As shown in Figure 1A, CsrA was not detected during the TP in WT cells, while it was highly accumulated in the Δ*clpP* mutant. During the RP, CsrA were detected in both WT and Δ*clpP*, with no significant difference between them. These data indicate that ClpP is involved in the temporal regulation of CsrA.

Next, we determined whether CsrA is a substrate of ClpP. ClpP^trap^ is a proteolytically inactive form of ClpP that will retain but not degrade substrates translocated into its proteolytic chamber (Flynn et al., 2003; Neher et al., 2006; Feng et al., 2013). To generate the ClpP^trap^, the active site (serine 110) of ClpP was replaced with an alanine (S110A) (Supplementary Figure S3A). The plasmids expressing His-tagged ClpP^wt^ and ClpP^trap^ were transformed into Δ*clpP* to create Δ*clpP*/p*clpP*^*wt*^ and Δ*clpP*/p*clpP*^*trap*^, respectively (Supplementary Figure S3A). Expression of the His-tagged recombinant ClpP proteins was verified in each strain (Supplementary Figure S3B). Growth curves were similar for Δ*clpP*/p*clpP*^*wt*^ and WT strains (Supplementary Figure S3C) as well as Δ*clpP*/p*clpP*^*trap*^ and Δ*clpP* (Supplementary Figure S3D). The heterologous level of intact or mutated *clpP* does not interfere with *Legionella* growth. Thus, we have successfully constructed strains to screen the substrates of ClpP.

Substrates captured inside the proteolytic barrel were co-purified along with the His-tagged ClpP complex and identified by mass spectrometry as previously described (Feng et al., 2013). We found that CsrA was detected in Δ*clpP*/p*clpP*^*trap*^, but not in Δ*clpP*/p*clpP*^*wt*^ (Figure 1B, Supplementary Figure S4), indicating that CsrA is a substrate of ClpP. Taken together, these data demonstrated that CsrA is temporally expressed during the life cycle and its regulation is dependent on ClpP.

### The Protein Level of CsrA Is Critical for Transition of *L. pneumophila* From the TP Into the RP

Because *csrA* is an essential gene, conditional and partial mutants are strongly attenuated for growth (Molofsky and Swanson, 2003; Sahr et al., 2017). Moreover, CsrA controls its own expression in a regulatory feedback loop (Sahr et al., 2009, 2017; Yakhnin et al., 2011). These attributes make the study of CsrA regulation difficult, but also crucial. To sidestep these issues, we constructed plasmids pJB908-*csrA* to express CsrA and pJB908-*gfp* to express GFP, both under control of the *mip* promoter (Supplementary Figure S5A). The transcriptional levels of *mip* and *csrA* are consistent in the RP and the TP of *L. pneumophila* (Bruggemann et al., 2006; Faucher et al., 2011). The plasmids were transformed into WT and Δ*clpP* to create WT/p*csrA*, Δ*clpP*/p*csrA*, WT/p*gfp*, and Δ*clpP*/p*gfp* (Supplementary Figure S5A).

**Figure 2:**
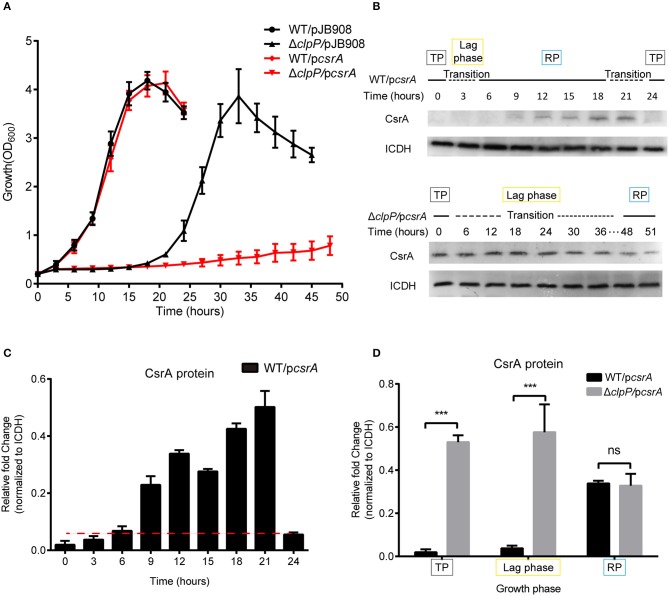
Accumulation of CsrA due to loss of ClpP regulation delays the transition of *L. pneumophila* from the TP into the RP. **(A)** Growth curves of *L. pneumophila* wild-type strain WT (•), the *clpP* deletion mutant Δ*clpP* (▴), WT with *csrA* expression (WT/p*csrA*) (▼), Δ*clpP* with *csrA* expression (Δ*clpP*/p*csrA*) (◆). For negative controls, pJB908 vector was electroporated into WT and Δ*clpP* to create WT/pJB908, Δ*clpP*/pJB908, respectively. Bacterial strains in TP (OD_600_ = 3.0–3.5) were grown in AYE medium at 37°C and samples were taken every 3 h for determination of optical density at 600 nm. **(B)** Ectopic protein levels of CsrA during the bacterial growth period. Bacterial whole-cell lysates from WT/p*csrA* and Δ*clpP*/p*csrA* were prepared and an immunoblot of CsrA was probed with an anti-His tag antibody. ICDH was measured as a loading control. RP refers to the exponential growth of bacteria in AYE broth, and TP refers to the period approximately 6 h after the cessation of growth. Lag phase refers to the transition from transmissive phase to replicative phase in rich medium. **(C)** Ectopic protein levels of CsrA in WT/p*csrA* calculated by ImageJ at the indicated time points. **(D)** Relative protein levels of CsrA in the RP, lag phase, and TP of WT/p*csrA* and Δ*clpP*/p*csrA* calculated by ImageJ. Bacterial cells in the RP were harvested at an OD_600_ of 0.7–1.0 and those in the TP were harvested approximately 6 h after the cessation of growth. Data represent mean ± SD derived from three independent experiments. ***p < 0.001 were identified by GraphPad Prism.

Bacterial inoculum from the TP culture was used to measure the impact of CsrA on the life cycle. Compared to WT/pJB908 and Δ*clpP*/pJB908, ectopic CsrA did not affect the growth of WT, but significantly prolonged the lag phase of Δ*clpP* (*p* < 0.001) and weakened its proliferation (Figure 2A). Meanwhile, GFP control did not affect the growth of either WT or Δ*clpP* (Supplementary Figure S6), indicating that the effect of CsrA on the growth of Δ*clpP* is indeed due to the lack of the regulation of CsrA by ClpP, rather than non-specific stress from protein accumulation.

Lysates were prepared from the indicated time points (Figure 2A) and analyzed by Western blotting. In WT/p*csrA*, CsrA was undetected during the TP and the transition from the TP to the RP, but was detected during the RP (Figure 2B), indicating that the level of CsrA is life-cycle-dependent (Figure 2C). In Δ*clpP*/p*csrA*, however, CsrA was detected in both the TP and throughout the transition phase (Figure 2B). Liquid culture observation and quantitative Western blotting analysis showed that, compared to WT/p*csrA*, the protein level of CsrA in the *clpP* mutant significantly increased during the TP and the lag phase, while it was identical during the RP (Figure 2D), indicating that the degradation of CsrA is ClpP-dependent and occurs during the TP. Meanwhile, the upregulation of the protein level of CsrA in WT/p*csrA* during the RP indicates that the degradation of CsrA *via* ClpP is temporally regulated. In the negative control, GFP continuously accumulated and the protein levels were consistent in WT/p*gfp* and Δ*clpP*/p*gfp* at the same growth phase (Supplementary Figures S7A–C), confirming that CsrA is regulated by ClpP. Our analysis indicated that ClpP is expressed throughout the life cycle of *L. pneumophila* (Supplementary Figure S8), indicating that its protein hydrolysis function is necessary during the biphasic life cycle. These data demonstrate that the accumulation of CsrA delays the transition of *L. pneumophila* from the TP into the RP, and that the protein level of CsrA is life-cycle-dependent and temporally regulated *via* ClpP during the transition between the replicative and transmissive forms.

### Observation of Morphology Indicates That Accumulation of CsrA During the TP Affects the Transition Into the RP

**Figure 3:**
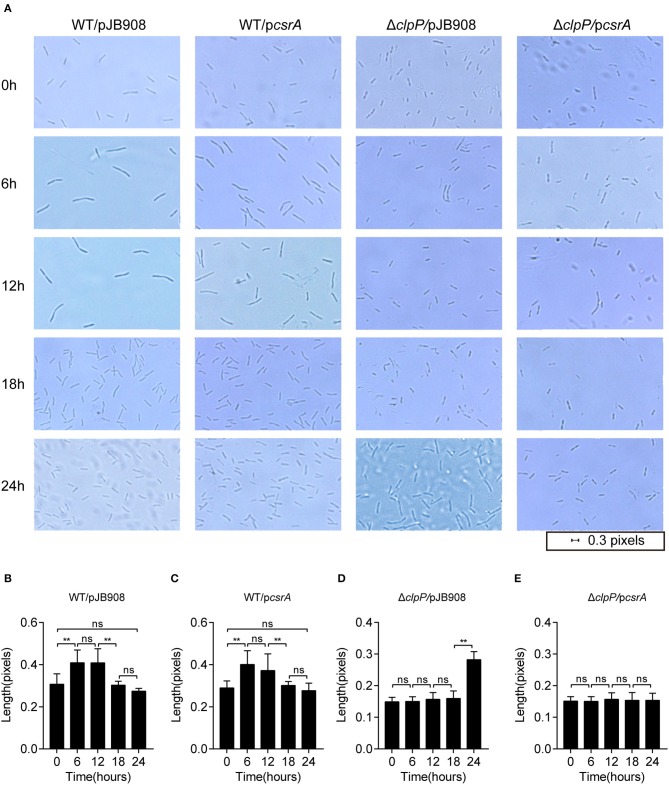
Morphological observation shows that the ClpP-dependent level of CsrA affects entry into the RP. **(A)** Bacterial morphology of *L. pneumophila* observed with 100× magnification under oil at the indicated time points. The indicated strains in the TP were inoculated into AYE media to the same initial OD_600_ of 0.2 with shaking at 37°C. **(B–E)** Cell length of WT/pJB908 **(B)**, WT/p*csrA* **(C)**, Δ*clpP*/pJB908 **(D)**, and Δ*clpP*/p*csrA* **(E)** was calculated with ImageJ at the indicated time points. Data represent mean ± SEM derived from three independent experiments. **p < 0.01 were identified by GraphPad Prism. ns means no difference from the wild type.

*L. pneumophila* cells alternate between different morphogenetic forms, including the slender rods in the RP and the short rods in the TP during its biphasic life cycle (Oliva et al., 2018). The morphological changes of the indicated strains were analyzed during their life cycle (Figure 3). Cells were observed under 100× magnification, and their lengths were measured with ImageJ at different time points during growth. In the WT strains with or without ectopic expression of *csrA*, the cells showed typical morphology corresponding to each growth phase (Figure 3A, row1 and 2, Figures 3B,C). However, compared to Δ*clpP*, the changes of cell length in the Δ*clpP* with accumulation of CsrA occurred much later throughout the indicated time points (Figure 3A, rows 3 and 4, Figures 3D,E), indicating that the accumulation of CsrA due to the loss of the regulation of ClpP (Figure 1B) blocked entry into the RP.

The growth phenotypes were characterized by measuring the transcriptional levels of the RP genes (*mip* and *secE*) and the TP genes (*flaA* and *fliA*) (Bruggemann et al., 2006) by qRT-PCR. The transcriptional level of life-cycle-dependent genes demonstrates that Δ*clpP* mutant remains in the TP during the prolonged lag phase (Figure 3B; Supplementary Figure S9A). Expectedly, the TP genes were upregulated while the RP genes were downregulated during the prolonged lag phase in Δ*clpP*/p*csrA* (Supplementary Figure S9B). The transcription levels of the four genes were determined in Δ*clpP*/pJB908 and Δ*clpP*/p*csrA* at time points of 0, 6, 12, and 18 h. Compared to Δ*clpP*, TP genes were highly upregulated while RP genes were suppressed with ectopic expression of *csrA* in Δ*clpP* (Supplementary Figure S9C). This indicates that the prolonged lag phase of Δ*clpP*/p*csrA* is associated with the accumulation of CsrA (Figure 2B). In conclusion, cell morphology is in agreement with the molecular reality during life cycle transition, indicating that the CsrA control *via* ClpP is critical for life cycle transition in *L. pneumophila*.

### Accumulation of CsrA During the TP Reduces the Viability of *L. pneumophila* in the Amoebae *Acanthamoeba castellanii*

**Figure 4:**
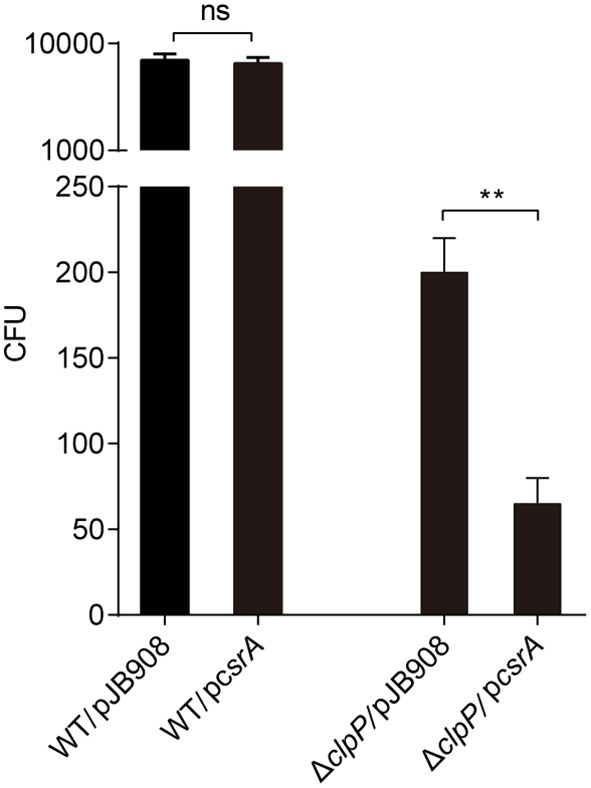
Viability of strains in TP with accumulated CsrA is impaired within the amoebae *A. castellanii* at the early post infection stage. *A. castellanii* were infected with TP-phase strains WT/pJB908, Δ*clpP*/pJB908, WT/p*csrA* and Δ*clpP*/p*csrA* at an MOI = 10. pJB908 vector can express the thymine required for growth of bacteria *in vivo*. Thirty minutes post infection, extracellular bacteria were removed by washing with warm HL5 medium three times. Infected amoebae cells were lysed after 90 min and intracellular bacteria were quantified by determining the CFU. Each time point represents the mean ± SD from three independent experiments. The quantitative data were analyzed using two-way analysis of variance (ANOVA) test by GraphPad Prism. The values that are significantly different are indicated by a bar and asterisk as follows: **p < 0.01. ns means no difference from the wild type.

To investigate whether the accumulation of CsrA affects the bacterial infectivity to host cells, *L. pneumophila* strains in TP were exposed to amoebae *A. castellanii* and the co-cultures were maintained for 2 h. Then, the extracellular bacteria were cleared and the amoebae were lysed to release *L. pneumophila* and calculate colony-forming units (CFU) of the infectious bacteria. The survival capability of WT/p*csrA* was similar to WT after phagocytosis (Figure 4). However, the survival capability of Δ*clpP*/p*csrA* was significantly (*p* < 0.01) lower than that of Δ*clpP* harboring the empty vector (Figure 4). These results indicate that the protein level of CsrA is important for the viability of *L. pneumophila* after phagocytosis.

### The Transcription of CsrA Is Temporally Regulated in a ClpP-Dependent Manner

**Figure 5:**
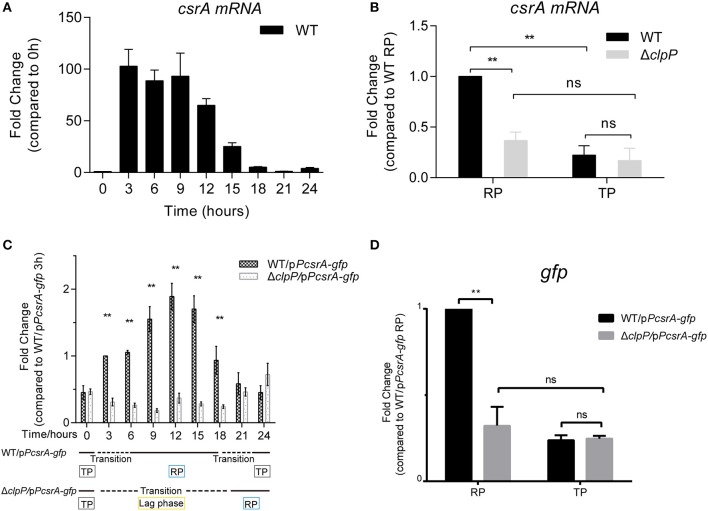
The transcription of *csrA* is temporally regulated by ClpP during the transition between the replicative and transmissive forms. **(A)** Transcriptional profile of *csrA* of WT. Total RNA was prepared from WT at the indicated time points. The transcriptional levels at 0 h were normalized to 1.0. **(B)** Comparison of the transcriptional levels of *csrA* in WT and Δ*clpP*. Bacterial cells in the RP were harvested at an OD_600_ of 0.7–1.0 and those in the TP were harvested approximately 6 h after the cessation of growth, and the total RNA was prepared. The transcriptional levels of WT at RP were normalized to 1.0. Data represent mean ± SD derived from three independent experiments. **p < 0.01 were identified by GraphPad Prism. **(C)** Transcriptional profiles of *gfp* under the control of *csrA* promoter during the life cycle. Total RNA was prepared from WT and Δ*clpP* harboring a p*PcsrA–gfp* reporter plasmid at the indicated time points. Transcriptional levels in WT/p*PcsrA-gfp* at 3 h were normalized to 1.0. Data represent mean ± SD derived from three independent experiments. **p < 0.01 were identified by GraphPad Prism. **(D)** Relative transcriptional levels of *gfp* in the RP and TP calculated by ImageJ. Bacterial cells in the RP were harvested at an OD_600_ of 0.7–1.0 and those in the TP were harvested approximately 6 h after the cessation of growth. Data represent mean ± SD derived from three independent experiments. **p < 0.01 were identified by GraphPad Prism.

Both the endogenous (Figure 1A) and heterologous protein levels of CsrA (Figures 2B,C) are regulated by ClpP throughout the life cycle of *L. pneumophila*. We investigated whether the regulation was under transcriptional control by ClpP as well. We found that the transcriptional level of *csrA* was upregulated in WT upon entry into the RP but was repressed during the TP (Figure 5A), indicating that the transcription of *csrA* is also temporal. We used qRT-PCR for transcriptional analysis of *csrA* in WT and Δ*clpP*. Compared to WT, the transcriptional level of *csrA* in Δ*clpP* significantly decreased during the RP, while it was identical during the TP (Figure 5B), suggesting that the transcription of *csrA* is also life-cycle-dependent and temporally regulated in a ClpP-dependent manner.

To rule out the influence of self-regulated CsrA, a vector in which *gfp* is expressed under the control of the *csrA* promoter was transformed into WT and Δ*clpP* (Supplementary Figure S5B). The expression of *gfp* did not affect the growth of WT or Δ*clpP* (Supplementary Figure S6). As shown in Figure 5C, the transcriptional level of *gfp* in Δ*clpP* is consistent throughout the life cycle, while it is life-cycle-dependent in WT. This result confirms that *csrA* transcription is temporally regulated in a ClpP-dependent manner. Quantitative analysis further supported this conclusion because the *gfp* level in Δ*clpP* was significantly downregulated compared to that in WT during the RP, but was identical during the TP (Figure 5D). Taken together, these data indicate that the transcription of CsrA is also temporally regulated in a ClpP-dependent manner.

### IHFB Binds Directly to the *csrA* Promoter Region

**Figure 6:**
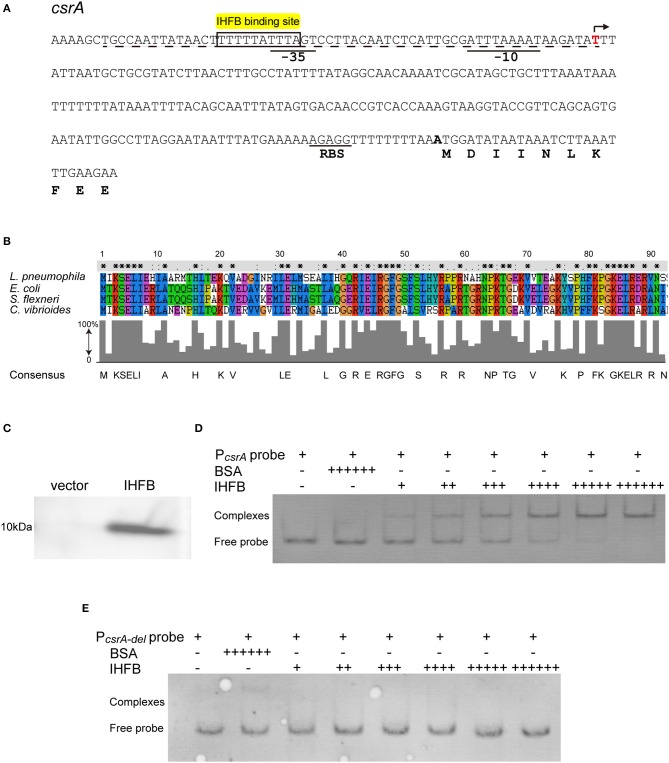
IHFB binds directly to the regulatory region of *csrA*. **(A)** Map of the promoter regions of *csrA* and positions of putative IHF binding sites. The transcriptional start site is indicated by an angled arrow. Possible −10 site, −35 site, and RBS site sequences are underlined. Predicted IHFB binding sites are labeled in yellow. The dashed line indicates the 60 bp DNA fragment that is deleted in the “P*csrA-del*” probe. Bioinformatics analysis was performed using Softberry software. **(B)** Sequence alignment of the putative IHFB from *L. pneumophila* with other prokaryotic IHF proteins. Numbers indicate the positions of amino acids in the sequences. Identical or similar residues are labeled with asterisks or periods, respectively. **(C)** Immunoblot of IHFB expressed in the BL21 strain of *E. coli*. The coding regions of IHFB were PCR amplified and fused to the expression vector pET-28a by the Gibson assembly method. The fusion gene constructs were transformed into *E. coli* strain BL21. Expression of IHFB was induced by IPTG to a final concentration of 10 μM. Proteins extracted from equivalent numbers of recombinant bacteria (1 × 10^9^ cells) were loaded onto an SDS-PAGE gel and IHFB was detected using an anti-His tag antibody. **(D,E)** EMSA analysis of *in vitro* binding of IHFB to the promoter region of *csrA* (P*csrA* probe) **(D)** and the promoter region deleted 60 bp upstream from the transcriptional start site of *csrA* (P*csrA-del* probe) **(E)**. An equivalent amount of the *csrA* probe DNA (200 ng) was added to every lane. BSA is used as a negative control for normalization, in which the amount is excessive (+ + + + ++). The first two lanes contain no IHFB (–) and the amount of IHFB is gradually increased (+). Each + sign indicates 200 ng of protein.

The realization that *csrA* is regulated by ClpP raises the possibility that the expression of *csrA* is controlled by an unknown transcriptional inhibitor that is degraded by the ClpP-mediated pathway. To test this possibility, we performed bioinformatic analysis of the upstream region of the *csrA* operon and found an IHF binding site (Figure 6A). IHF is a global transcriptional regulator reported in *Escherichia coli, Shigella flexneri*, and *Caulobacter* (Craig and Nash, 1984; Gober and Shapiro, 1990; Porter and Dorman, 1997; Ali Azam et al., 1999; Goodman et al., 1999). Alignment analysis of the known IHF sequences identified the highly conserved IHFB protein as the candidate regulator in *L. pneumophila* (Figure 6B).

To determine if the IHFB protein binds to the *csrA* promoter region, we performed DNA binding electrophoretic mobility shift assay (EMSA). First, the *ihfB* gene was fused with a His-tag to produce a recombinant fusion protein (Figure 6C). Then, serial concentrations of the fusion protein were incubated with the 600-bp-long DNA fragment upstream of the *csrA* start codon. The same fragment with a 60-bp deletion in the 5′ noncoding region was used as a control. As shown in Figure 6D, recombinant IHFB produced a gel shift with the full length of the DNA fragment in a concentration-dependent manner, whereas IHFB incubated with the truncated fragment did not produce a gel shift (Figure 6E). These data indicate the direct binding of IHFB to the promoter region of *csrA*.

### IHFB Is a Transcriptional Inhibitor That Regulates the Temporal Transcription of *csrA*

**Figure 7:**
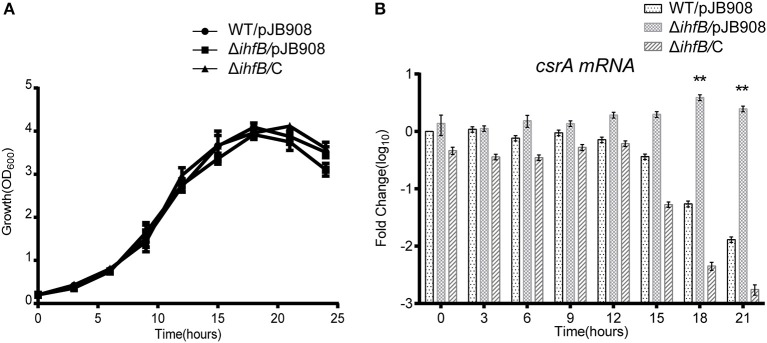
IHFB is a transcriptional inhibitor of *csrA*. **(A)** Growth curves of WT (•), *ihfB* deletion mutant Δ*ihfB* (■), and the complemented strain Δ*ihfB*/C (▴). For negative controls, pJB908 vector was electroporated into WT and Δ*ihfB* to create WT/pJB908 and Δ*ihfB*/pJB908, respectively. Bacterial inoculum from the TP culture was inoculated into AYE media to the same initial OD_600_ of 0.2 at time zero. Bacterial cells were grown in AYE medium at 37°C and samples were taken every 3 h for determination of optical density at 600 nm. **(B)** Relative transcriptional profiles of *csrA* in WT, Δ*ihfB*, and the complemented strain Δ*ihfB*/C during the life cycle. Total RNA was prepared at the indicated time points. Transcriptional level of *csrA* was detected by qRT-PCR. The transcriptional levels of *csrA* in WT/pJB908 at 0 h were normalized to 0 by taking log10. Data represent mean ± SD derived from three independent experiments. **p < 0.01 were identified by GraphPad Prism.

Since transcription of *csrA* in WT is temporally regulated (Figures 5B,D) and IHFB binds directly to the promoter region of *csrA* (Figure 6), we hypothesized that IHFB is a transcriptional regulator of *csrA*. To test this, we investigated the effect of *ihfB* deletion on the transcription of *csrA*. An *ihfB* deletion strain (Δ*ihfB*) was constructed using a non-polar deletion strategy. The growth kinetics of the Δ*ihfB* mutant in AYE were similar to WT and *ihfB*-complemented strains (Figure 7A), indicating that IHFB is not essential for *L. pneumophila* growth *in vitro*. qRT-PCR results showed that the transcriptional level of *csrA* in the Δ*ihfB* mutant was persistent during the entire life cycle (Figure 7B), whereas in WT, it was downregulated during the TP (Figure 7B, time points 18 to 21). These results indicate that IHFB inhibits the transcription of *csrA* during the TP.

### IHFB Is Temporally Expressed During the Life Cycle and Is Degraded by ClpP During the RP

**Figure 8:**
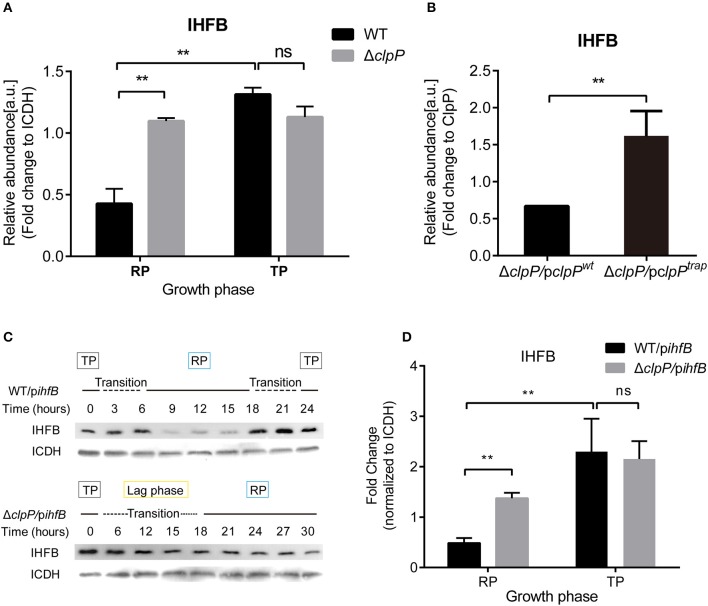
IHFB is temporally expressed and is degraded by ClpP during the RP. **(A)** The abundance of IHFB in WT and Δ*clpP* determined by proteomics analysis. WT and Δ*clpP* were cultured in fresh AYE medium at the same initial OD_600_ values. Bacterial cells in the RP were harvested at an OD_600_ of 0.7–1.0 and those in the TP were harvested approximately 6 h after the cessation of growth. Total proteins from indicated samples were extracted for proteomic analysis. Bacterial whole-cell lysates from WT and Δ*clpP* were prepared and identified by mass spectrometry. ICDH was measured as a loading control. **(B)** The abundance of IHFB in Δ*clpP*/p*clpP*^*wt*^ and Δ*clpP*/p*clpP*^*trap*^ were identified by ClpP^trap^ proteomic analysis. Bacterial cells in TP were harvested approximately 6 h after the cessation of growth. Whole-cell lysates from Δ*clpP*/p*clpP*^*wt*^ and Δ*clpP*/p*clpP*^*trap*^ were prepared and His-tagged ClpP proteins were purified by Ni-NTA affinity. Substrates captured inside the proteolytic barrel were co-purified along with the His-tagged ClpP complex and identified by mass spectrometry. ClpP was measured as a loading control. **(C)** IHFB is degraded by ClpP during the RP. Whole-cell lysates were prepared from equal amounts of cells of WT/p*ihfB* and Δ*clpP*/p*ihfB* at indicated time points, and an immunoblot of IHFB was performed using an anti-His tag antibody. ICDH was probed as a loading control. **(D)** Relative protein levels of IHFB in the RP and the TP calculated by ImageJ. Bacterial cells in the RP were harvested at an OD_600_ of 0.7–1.0 and those in the TP were harvested approximately 6 h after the cessation of growth. The quantitative data were analyzed using two-way analysis of variance (ANOVA) test by GraphPad Prism. The values that are significantly different are indicated by a bar and asterisk as follows: **p < 0.01.

We measured the protein level of IHFB during different growth phases using proteomic analysis of whole cell lysates obtained from cultures of WT and Δ*clpP* grown in liquid medium. By quantifying the IHFB-representative peptide (Supplementary Figure S10), we found that the protein level of IHFB in Δ*clpP* was significantly (*p* < 0.01) higher than WT during the RP, whereas the protein level of IHFB in Δ*clpP* was similar to WT during the TP (Figure 8A). These data indicate that endogenous IHFB is temporally expressed and that its protein level is regulated in a ClpP-dependent manner.

We applied ClpP trapping analysis to determine whether IHFB is a substrate of ClpP (Supplementary Figure S11). Using the protein level of ClpP as a reference, we identified that although IHFB was captured in both Δ*clpP*/p*clpP*^*wt*^ and Δ*clpP*/p*clpP*^*trap*^, significantly more IHFB was detected in Δ*clpP*/p*clpP*^*trap*^ than in Δ*clpP*/p*clpP*^*wt*^ (Figure 8B). We interpret that Δ*clpP*/p*clpP*^*wt*^ captures and degrades IHFB, whereas Δ*clpP*/p*clpP*^*trap*^ accumulates IHFB because it cannot degrade the captured IHFB. Our results suggest that IHFB is a substrate of ClpP. Thus, ClpP may directly control IHFB, the negative transcriptional regulator of *csrA*.

IHFB protein stability might also be regulated in a ClpP-dependent manner. To test this possibility, *ihfB* was fused with a C-terminal His-tag in plasmid pJB908-*ihfB*, which was transformed into WT and Δ*clpP* (Supplementary Figure S5C). The expression of *ihfB* did not affect the growth kinetics of WT or Δ*clpP* (Supplementary Figure S6). The protein levels of IHFB were detected by Western blotting using an anti-His antibody. As shown in Figure 8C, IHFB persistently accumulated in both strains during the TP and transition. However, the amount of IHFB in WT decreased significantly during the RP compared to Δ*clpP*, indicating that the protein level of IHFB is regulated in a ClpP-dependent manner. The GFP control continuously accumulated and the protein levels were consistent at the same growth phase between WT/p*gfp* and Δ*clpP*/p*gfp* (Supplementary Figures S7A–C). Quantitative analysis confirmed that the protein level of IHFB in Δ*clpP* was significantly (*p* < 0.01) higher than WT during the RP, but similar during the TP (Figure 8D).

### ClpP-Mediated Degradation of CsrA Protein During the TP Is IHFB-Independent

**Figure 9:**
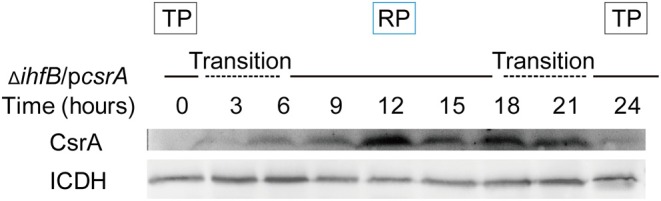
Expression of CsrA in Δ*ihfB* during the life cycle reveals that the degradation of CsrA during the TP is IHFB-independent. Bacterial whole-cell lysates were prepared from Δ*ihfB*/p*csrA* and an immunoblot of CsrA was performed using an anti-His tag antibody. ICDH was probed as a loading control.

We test whether the ClpP-dependent inhibition of *csrA* transcription by IHFB and the ClpP-mediated degradation of CsrA protein were independent processes. The plasmid pJB908-*csrA*, in which the expression of *csrA* is controlled by the *mip* promoter, was transformed into Δ*ihfB*, resulting in the strain Δ*ihfB*/p*csrA*. To ensure that IHFB did not affect the expression of the *mip* promoter-controlled gene, we used *gfp* under the control of *mip* promoter in WT and Δ*ihfB*. The result showed that the expression of *gfp* was consistent in both strains at the same growth phase (Supplementary Figures S7A,B,D). The expression profile of *csrA* was then measured in Δ*ihfB*/p*csrA*. Similar to the protein level of CsrA in WT (Figure 2B), in a new life cycle of Δ*ihfB*/p*csrA*, CsrA was undetected during the TP but was upregulated during the RP (Figure 9). This indicated that the protein level of accumulated CsrA was temporally regulated in the absence of *ihfB*. Combining with the result in Figure 2B, these data indicate that the ClpP-dependent transcriptional inhibition of *csrA* by IHFB does not affect the ClpP-mediated degradation of CsrA during the biphasic life cycle of *L. pneumophila*.

**Figure 10:**
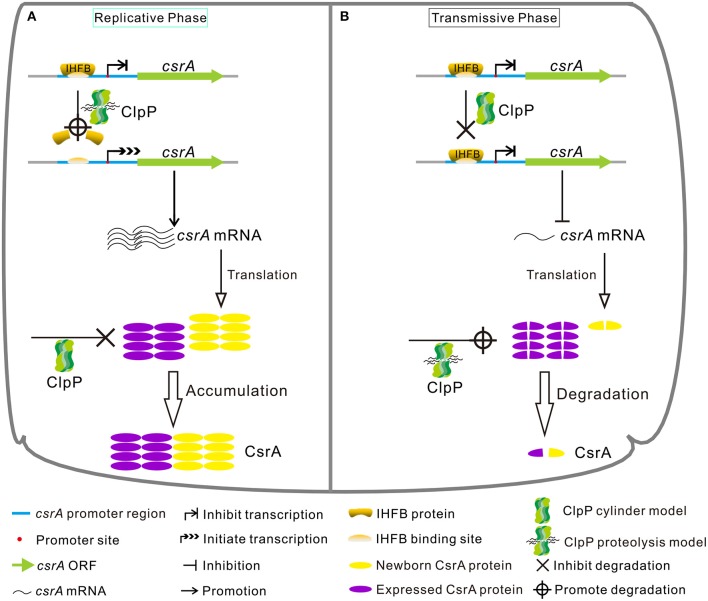
Model of regulatory cascade of the expression of CsrA during the biphasic life cycle of *L. pneumophila*. **(A)** During the replicative phase, the transcription of *csrA* increases due to the degradation of the transcription inhibitor IHFB *via* ClpP, while the CsrA protein is avoided to be degraded by ClpP *via* an unknown mechanism, resulting in the accumulation of CsrA. **(B)** During the transmissive phase, IHFB binds to inhibit the transcription of *csrA*, while the accumulated CsrA is degraded by ClpP, leading to the decrease of intracellular CsrA. See text for details.

In conclusion, our study demonstrates that the expression of CsrA is temporally regulated in a ClpP-dependent manner both at the protein and transcriptional levels during the biphasic life cycle of *L. pneumophila* (Figure 10). Specifically, during the RP (Figure 10A), the accumulation of CsrA is promoted in a ClpP-dependent manner by degrading the transcriptional inhibitor IHFB to promote the transcription of *csrA* and reducing the degradation of the CsrA protein. When nutrients become limiting (Figure 10B), the level of CsrA is decreased in a ClpP-dependent manner by ceasing the degradation of IHFB to inhibit the transcription of *csrA* and promoting the degradation of the accumulated CsrA.

## Discussion

To adapt to various harsh environmental conditions *in vitro* and in host cells, *L. pneumophila* adopts a biphasic life cycle, allowing it to switch between an RP and a transmissive/virulent phase, and exclusively expresses genes required for the transition (Molofsky and Swanson, 2004; Bruggemann et al., 2006). Although several regulators, such as LetA/S, RpoS, PmrA, CpxR, rsmX/Y/Z, and LqsR, that govern the *Legionella* life cycle have been characterized (Gal-Mor and Segal, 2003; Bachman and Swanson, 2004; Jacobi et al., 2004), the network involved in controlling the life cycle cascade is still incompletely understood. *L. pneumophila* CsrA is a pivotal repressor of transmission traits and activator of replication. It controls the switch from replicative to transmissive virulence phase of infection (Molofsky and Swanson, 2003). It has been demonstrated that CsrA-dependent repression of transmission traits is alleviated by the LetS/LetA TCS and that RsmY/Z link the LetS/LetA TCS and CsrA to the control of replication vs. transmission phases (Sahr et al., 2009). Though the majority of LetS/LetA-regulatory effects depend on RsmY/Z, regulation of several motility genes does not. Analysis of the transcriptional programs of the Δ*letA*, Δ*letS*, and Δ*rsmYZ* strains revealed that the switch to TP is partially blocked (Sahr et al., 2009). These data suggest that there may be other regulatory pathways involved in the regulation of CsrA. The results of this study indicate that the temporal expression of CsrA is dually regulated in a ClpP-dependent manner. This extends our understanding of how the bacteria manipulate their life cycle by using multiple strategies. Given that ClpP and CsrA are highly conserved in all *L. pneumophila* strains sequenced so far (Supplementary Figures S12, S13), the underlying mechanism of the life cycle control by CsrA and ClpP may be representative of the genus *Legionella* or Gram-negative bacteria in general.

Regulated proteolysis of native regulatory proteins is required for bacteria to maintain quality control and undergo cell-cycle progression, physiological transitions, and adaptations needed for survival and persistence (Mahmoud and Chien, 2018). In the aquatic dimorphic organism *Caulobacter crescentus*, ClpP regulates the swarmer-to-stalked transition (Joshi and Chien, 2016). *Bacillus subtilis* requires proteolysis by ClpXP to initiate a sporulation program from mature spores to dead spores (Tan et al., 2015). In *E. coli*, ClpP regulates the transition from logarithmic growth to stationary phase (Hengge, 2009). However, the stage of life cycle control that ClpP is involved in is still unclear. We found that the growth curve and bacterial morphology observed in the Δ*clpP* mutant revealed a prolonged lag phase compared to WT (Figures 2A, 3). Further detection of the transcriptional level of life-cycle-dependent genes demonstrated that the prolonged lag phase of the Δ*clpP* mutant remained in the TP (Figure 3B; Supplementary Figure S9A). Ectopic expression further revealed that intracellular accumulation of CsrA delayed the transition of *L. pneumophila* from the TP into the RP. Temporal expression of CsrA was dually regulated in a ClpP-dependent manner during the transition between the replicative and transmissive forms. Therefore, the growth defects of Δ*clpP* may be caused by insufficient control of CsrA. It is noteworthy that, because *csrA* is essential for *Legionella* growth, all studies on the regulation of *csrA* expression were performed using an ectopic expression strategy (Fettes et al., 2001; Sahr et al., 2009, 2017; Yakhnin et al., 2011), including the present study. Likewise, ClpP is essential for bacteria to acclimate to their niche for growth. For example, the loss of *clpP* in *Streptococcus pneumoniae*, cyanobacteria *Synechococcus*, and *Salmonella enterica* serovar Typhimurium is responsible for growth defects at reduced temperatures (Porankiewicz et al., 1998; Robertson et al., 2002; Knudsen et al., 2014) although the mechanism is unknown. Our finding that the regulation of CsrA by ClpP affects growth of *L. pneumophila* suggests a shared regulatory strategy by these bacteria.

The impaired growth of the *clpP* mutant *L. pneumophila* is associated with high levels of CsrA (Figures 2A,B, 3C,E, 4). This is different from *S*. *enterica* serovar Typhimurium in which the impaired growth at low temperature due to the *clpP* deletion is associated with high levels of stationary-phase-specific sigma factor RpoS (Knudsen et al., 2014). In addition, ClpP indirectly regulates CsrA through RpoS to reduce the virulence in *S. enterica* serovar Typhimurium (Knudsen et al., 2013). Previous work has shown increased expression of RpoS in an *L. pneumophila csrA* mutant (Forsbach-Birk et al., 2004), which is in line with the observations in *S. enterica* serovar Typhimurium (Knudsen et al., 2013). However, RpoS is not involved in the growth-phase-dependent resistance to stress in *L. pneumophila*. Rather, it likely regulates the genes that enable the bacteria to survive within protozoa (Hales and Shuman, 1999). Our data also show that the impaired growth of the *clpP* mutant of *L. pneumophila* is not associated with high levels of RpoS (data not shown), but with high levels of CsrA. These data suggest that ClpP is involved in the regulation of bacterial growth by a different pathway in *L. pneumophila* than in *S. enterica* serovar Typhimurium.

In addition to our finding that ClpP degrades CsrA, we revealed that the protein level of IHFB, the transcriptional inhibitor of CsrA, is also controlled by ClpP (Figure 8). Originally classified as an architectural protein in *E. coli*, IHF is also a transcriptional regulator that appears to be conserved in function, as homologs of IHF are involved in the regulation of gene expression in a number of closely and distantly related bacteria, including pathogens (Goosen and van de Putte, 1995; Fyfe and Davies, 1998; Dorman et al., 2001; Sieira et al., 2004; Mangan et al., 2006; Stonehouse et al., 2008; Perez-Rueda et al., 2009; Arvizu-Gomez et al., 2011). This suggests that IHF plays a role in virulence. For example, *ihf* in *C. crescentus* is required for temporal activation of flagellar genes during its life cycle and it promotes efficient chromosomal replication (Gober and Shapiro, 1990; Quon et al., 1996; Porter and Dorman, 1997; Siam et al., 2003). In *L. pneumophila*, although *ihfB* is not required for growth *in vitro* (Figure 7A), our unpublished data show that Δ*ihfB* mutants fail to grow in *A. castellani*, consistent with the finding that IHF is required for full virulence in amoebae (Morash et al., 2009). IHF is temporally expressed at minimal and maximal levels during the RP and TP, respectively (Morash et al., 2009), further suggesting a role for IHF in virulent phenotypes. Interestingly, IHF was also found to directly bind to and activate transcription of RsmY and RsmZ, two non-coding regulatory RNAs responsible for the de-repression of CsrA-repressed transcripts associated with the progression to the TP (Pitre et al., 2013). How ClpP is involved in temporal control of the IHF-sRNA-CsrA cascade is worthy of investigation in the future.

We performed a BLAST search of the *L. pneumophila* genome, and a set of potential IHF binding sites were identified in the upstream sequences of approximately 300 *L. pneumophila* genes (data not shown), including several regulators of post-exponentially expressed genes (*letA, letE, fleQ, rpoS*, and *ihfA*) (Morash et al., 2009) and effector-encoding genes. Moreover, CsrA is reported to control the expression of over 40 Dot/Icm substrates (Sahr et al., 2009, 2017) and is essential for intracellular growth (Morash et al., 2009). Lack of intracellular growth and infection efficiency of *L. pneumophila* in *A. castellanii* were observed in Δ*clpP* mutants (Figure 4), Δ*ihfB* mutants, and Δ*csrA* mutants (Forsbach-Birk et al., 2004; Morash et al., 2009; Li et al., 2010). These results indicate that the action of ClpP on CsrA and IHFB may play a more integral role in the regulation of the life cycle and virulence of *L. pneumophila*.

Based on our results and previous studies, we propose a model for the network of the life cycle controlled by CsrA, whose expression is regulated simultaneously by ClpP and IHFB (Figure 10). When bacteria are in a nutrient-rich environment, ClpP promotes the degradation of the response regulator CsrA (Figures 1, 2A,B). Therefore, *L. pneumophila* can normally enter the RP from the TP, initiating a new cycle. During the RP (Figure 10A), ClpP promotes the expression of CsrA by reducing the amount of the transcriptional inhibitor IHFB while ceasing degradation of CsrA protein. When nutrients become limiting (Figure 10B), ClpP promotes accumulation of IHFB to inhibit the transcription of *csrA*, and simultaneously promotes the degradation of accumulated CsrA to govern the transition from the RP to the TP. Overall, CsrA likely acts as a biphasic switch during the *Legionella* life cycle. It is finely regulated at dual levels to achieve control of metabolism/replication, motility, and virulent traits of *L. pneumophila*.

## Materials and Methods

### Bacterial Strains, Plasmids, Primers, and Media

The bacterial strains, plasmids, and primers used in this work are listed in Supplementary Tables S1, S2, respectively. All *L. pneumophila* strains were cultured on buffered charcoal yeast extract (BCYE) plates, or in N-(2-acetamido)-2-aminoethanesulfonic acid (ACES)-buffered yeast extract (AYE) medium, supplemented with thymidine (100 μg/ml) (Feeley et al., 1979) when required. *E. coli* DH5α and *E. coli* BL21(DE3), used as host strains for cloning strategies and recombinant protein expression, respectively, were grown in Luria-Bertani (LB) broth and agar at 37°C. For liquid culture, AYE broth was inoculated with TP bacteria grown in the previous cycle to a final OD_600_ of 0.2 and incubated at 37°C with vigorous shaking. RP bacteria were harvested at an OD_600_ of 0.7–1.0 and TP bacteria were harvested approximately 6 h after the cessation of growth, which is at an approximate OD_600_ of 3.0–3.5. Ampicillin (amp) was added to a final concentration of 100 μg/ml, kanamycin (kan) to 50 μg/ml, chloramphenicol (cm) to 34 μg/ml, and IPTG to 10 μM for *E. coli*, and chloramphenicol to 5 μg/ml for *L. pneumophila*. *A. castellanii* (ATCC 30234) was grown in proteose yeast extract glucose medium (PYG) at 30°C (Segal and Shuman, 1999). To ascertain CFU, serial dilutions of bacteria were incubated on BCYE for 4 days and resultant colonies were counted. Bacto yeast exact and proteose peptone were obtained from Becton Dickinson Biosciences. All other reagents were from Sigma Co., unless specified otherwise. All primers were synthesized by Ruibiotech Co., China. All restriction enzymes were purchased from New England Biolabs. All DNA cloning was carried out in the *E. coli* DH5a strain using standard molecular techniques. The protein concentration was determined using Bradford's protein assay reagent (Bio-Rad).

### Construction of Mutants and Plasmids

*ihfB* deletion strain was constructed by utilizing an in-frame gene replacement suicide vector (pBRDX) strategy (LeBlanc et al., 2008). Briefly, upstream and downstream flanking sequences of *ihfB* were amplified by PCR using the PΔ*ihfB*-F1/PΔ*ihfB*-R1 and PΔ*ihfB*-F2/PΔ*ihfB*-R2 primer pairs, respectively. The PCR products were mixed and then used as templates for the subsequent fusion PCR using the PΔ*ihfB*-F1/PΔ*ihfB*-R2 primers. Fusion PCR products were digested with B*gl*II and B*amH*I and sub-cloned into the pBRDX vector, creating pBRDXΔ*ihfB*. Then, pBRDXΔ*ihfB* was introduced into the WT strain by electroporation, and chloramphenicol^R+^ colonies were selected on BCYET-Cm plates. Transformants were inoculated into AYET and then incubated on BCYET containing 10% sucrose for 3 days at 37°C to select for strains devoid of the vector backbone. Positive colonies (Δ*ihfB*) were confirmed by PCR and sequencing.

### Complementation Assay

For complementation experiments, a RSF1010 pKB5-derived vector pJB908 was utilized as the cloning backbone (Sexton et al., 2004). To construct the complementing strain of *ihfB, ihfB* gene and its promoter region were amplified by PCR with the P*ihfB*-CF/P*ihfB*-CR primer pair and cloned into pJB908. The resulting plasmid pJB908-*ihfB* was electroporated into Δ*ihfB* to create the complemented strain Δ*ihfB*/C. For negative controls, pJB908 vector was electroporated into WT, Δ*clpP*, and Δ*ihfB* to create WT/pJB908, Δ*clpP*/pJB908, and Δ*ihfB*/pJB908, respectively.

### DNA Library Preparation and Whole-Genome Sequencing

For whole-genome sequencing, genomic (g) DNA of WT (LP02) and Δ*clpP* (XP02) were prepared from bacterial cultures using the Bacterial DNA kit (Omega Co.) and was used to construct gDNA library for genomic sequencing. Paired-end sequences and a read length of 100 bases were obtained from an Illumina HiSeq 2500. Sequence reads were mapped to a reference genome using genome alignment software BWA (Li and Durbin, 2009); single nucleotide polymorphisms (SNPs) and Insertion & Deletion (InDel) were searched using GATK (McKenna et al., 2010), and detection of all potential chromosome structural variation (SV) site in the whole genome was carried out by chromosome structure variation analysis software DELLY (Rausch et al., 2012) (Annoroad, China).

### *In vivo* Trapping of ClpP Substrate

The ClpP trapping system was constructed according to the previous report with minor modification (Feng et al., 2013). Briefly, to generate the ClpP^trap^, the active site (serine 110) of ClpP was replaced with an alanine (S110A). The plasmids expressing His-tagged ClpP^wt^ and ClpP^trap^ were transformed into Δ*clpP*, respectively, to create Δ*clpP*/p*clpP*^*wt*^ and Δ*clpP*/p*clpP*^*trap*^. The Δ*clpP*/p*clpP*^*wt*^ and Δ*clpP*/p*clpP*^*trap*^ strains in TP were grown in 100 ml of AYE at 37°C to an OD_600_ of 0.2. To screen accumulated substrates of ClpP^trap^ during the whole life cycle, bacterial whole-cell lysates from Δ*clpP*/p*clpP*^*wt*^ and Δ*clpP*/p*clpP*^*trap*^ in the TP (harvested approximately 6 h after the cessation of growth) were prepared and His-tagged proteins were purified with Ni-NTA affinity column (GE Healthcare) following the manufacturer's instructions. Substrates captured inside the proteolytic barrel were co-purified along with the His-tagged ClpP complex and identified by mass spectrometry to identify substrates of ClpP in the WT background. ClpP (Protein Accession: Q5ZUD9) was calculated as a loading control because the amount of ClpP determines how much the substrate is bound.

### Proteomic Analysis (LC-MS)

For each sample, 100 μg of protein was reduced with 10 mM dithiothreitol (DTT) at 37°C for 45 min and iodoacetamide (IAM) was then added to a final concentration of 15 mM, with incubation at room temperature for 1 h in the dark. The samples were then diluted with 100 mM ammonium bicarbonate buffer and digested with trypsin (1:50, trypsin/lysate ratio) for 16 h at 37°C. Digests were centrifuged through 3-kDa filter tubes so that only digested peptides can go through. Peptide concentrations were determined with a modified Lowry Protein Assay Kit (Sangon Biotech. Co.). Twenty micrograms of peptides was desalted on Pierce C18 Spin Columns (Thermo Fisher Scientific, Co.) according to the manufacturer's instructions. Peptides were analyzed with the Q Exactive HF-Orbitrap MS (Thermo Fisher Scientific, Co). For each sample, the same amounts of peptides from total protein were separated on the analytical column with a 70-min linear gradient at a flow rate of 400 nl/min (0–3% B in 3 min; 3–8% B in 4 min; 8–32% B in 44 min; 32–99% B in 5 min; 99% B for 4 min, 3% B for 10 min). The spectra were acquired in the positive ionization mode by data-dependent methods consisting of a full MS scan in high mass accuracy FT-MS mode at 60,000 resolutions, with the precursor ion scan recorded over the m/z range of 350–1500. Database searching of all LC-MS/MS raw files was performed in Proteome Discoverer 2.2 (Thermo Fisher Scientific, Co). MASCOT 2.2.4 and SEQUEST were used for database searching against the Uniprot *L. pneumophila* database (*L. pneumophila* subsp. pneumophila strain Philadelphia 1/ATCC 33152/DSM 7513 proteome, last modified: October 26, 2018; 2930 proteins). Proteomics analysis of the peptide data of CsrA (Protein Accession: Q5ZV47) and IHFB (Protein Accession: Q5ZRC7) in the RP and TP of WT and Δ*clpP* are shown in Supplementary Table S3; ICDH (Protein Accession: Q5ZXB6) was calculated as a loading control. Proteomics analysis of the peptide data of CsrA and IHFB purified by the ClpP trapping system is shown in Supplementary Table S4; ClpP (Protein Accession: Q5ZUD9) was calculated as a loading control.

### Construction of the Plasmids That Ectopically Express CsrA, IHFB, and GFP

To avoid the interference of transcriptional regulation of self-promoter, the plasmids for ectopic expression of CsrA, IHFB, and GFP were constructed. To this end, the sequence of *mip* promoter region and *csrA* gene was amplified by PCR using the P*pmip*-F/P*pmip*-R1 and P*csrA*-F/P*csrA*-R primer pairs. The PCR products were mixed and then used as templates for the subsequent fusion PCR using the P*pmip*-F/P*csrA*-R primers. Fusion PCR products were digested with S*ac*I and S*ph*I and sub-cloned into the pJB908 vector, creating plasmid pJB908-*csrA*. Likewise, the same strategy was employed to create plasmid PJB908-*ihfB* using primer pairs P*pmip*-F/P*pmip*-R2 and P*ihfB*-F/P*ihfB*-R and plasmid PJB908-*gfp* using primer pairs P*pmip*-F/P*pmip*-R3 and P*mgfp*-F/P*mgfp*-R. A hexa-histidine tag was added to the C-terminus of the protein in both the resulting recombinant plasmids pJB908-*csrA* and PJB908-*ihfB* during cloning. The resultant plasmid pJB908-*csrA* was electroporated into WT, Δ*clpP*, and Δ*ihfB* to create strain WT/p*csrA*, Δ*clpP*/p*csrA*, and Δ*ihfB*/p*csrA*, respectively. The resultant plasmid pJB908-*ihfB* was electroporated into WT and Δ*clpP* to create strain WT/p*ihfB* and Δ*clpP*/p*ihfB*. The resultant plasmid pJB908-*gfp* was electroporated into WT, Δ*clpP*, and Δ*ihfB* to create strain WT/p*gfp*, Δ*clpP*/p*gfp*, and Δ*ihfB*/p*gfp*, respectively.

### Growth Curve Assay and Bacterial Morphology Observation

Fresh *L. pneumophila* cells were inoculated into 5 ml of AYE(T) medium and were cultured to the TP at 37°C. Then, the cultures were transferred into 50 ml of AYE in flasks, incubated to the TP, and then diluted into new flasks to similar optical densities at an approximate OD_600_ of 0.2 at time zero. Cultures were grown at 37°C with shaking. To measure the growth curve, 1 ml of the cells was sampled every 3 h for measurement of absorbance at 600 nm. Bacterial cells for morphological observation were sampled at 0, 6, 12, 18, and 24 h after *L. pneumophila* cells were transferred into 50 ml of AYE. Light microscopic images of *Legionella* cells were captured at a 100-fold oil microscope using Leica LAS-EZ optical microscopy equipped with a camera. The length of the cells was presented by ImageJ. At least three sections of each sample were photographed, and one typical photograph was selected to represent. To ensure conformity, multiple replicates on different days were examined.

### Fluorescence Intensity Analysis of *mip* Promoter

The pJB908-*gfp* plasmid was transferred into the WT strain and the *clpP* mutant Δ*clpP*, respectively, to construct WT/p*gfp* and Δ*clpP*/p*gfp* strains. The culture operation of the bacteria is the same as the experimental procedure of growth curve assay. Bacteria were collected at OD_600_ of 1.0, 2.0, 3.0, and 4.0, respectively, from the liquid AYE for fluorescence intensity analysis. After the analysis of fluorescence intensity, the bacterial cells were plated on AYE plates from each period to detect bacterial viability. Bacterial cells were centrifuged at 5°C and 4,000× g. The cell pellets were resuspended in 20 ml of PBS buffer and were centrifuged at 4,000× g for 5 min at 4°C. This step was repeated once to ensure that the medium is completely removed. Then, the pellets were resuspended with PBS buffer and the cell suspension concentration was adjusted to approximately 10^8^ bacteria/ml with a spectrophotometer. The fluorescence intensity of the excitation fluorescence spectrophotometer was 488 nm, the absorption wavelength was 507 nm, and the fluorescence intensity was measured using PBS buffer as a blank control.

### RNA Isolation, cDNA Preparation, and qRT-PCR

RNA for real-time quantitative PCR (qRT-PCR) was prepared using an Eastep® super Kit following the manufacturer's protocols (Promega Co.) and treated with DNase I according to the manufacturer's instructions (Promega Co.) prior to cDNA preparation. cDNA was prepared using GoScript™ reverse transcription system as described by the manufacturer (Promega Co.). qRT-PCR was performed in a 20-μl reaction volume using an Applied Biosystems Step One Plus 96-well reverse transcription-PCR system with Power SYBR Green PCR Master Mix following the manufacturer's instructions (Applied Biosystems Co.). 16S rRNA was used as the reference sample in all comparative threshold cycle (ΔΔCT) experiments. All qRT-PCR primers were tested for amplification efficiency. qRT-PCR data were analyzed using Step One System software and GraphPad Prism. Primers used in qRT-PCR experiments are shown in Supplementary Table S2. All analysis was performed in biological triplicate.

### Bacterial Infectivity in *A. castellanii*

The *A. castellanii* cells were seeded onto a 24-well plate (5 × 10^5^ per well) and allowed to adhere for 2 h prior to infection. *L. pneumophila* cells were grown for 20 h in AYE broth at 37°C with shaking, diluted in HL5, and were used to infect amoebae at an MOI of 10. Thirty minutes post infection, extracellular bacteria were removed by washing three times with warm HL5 medium (Tiaden et al., 2007). At the indicated time points, culture supernatant was removed and the amoebae cells were lysed with 0.04% Triton. The supernatant and the lysates were combined, and serial dilutions were prepared and aliquots were plated on BCYE plates for CFU counting (Al-Khodor et al., 2008). All experiments were performed in triplicate at 30°C.

### Protein Isolation and Western Blotting

Total cell extracts of *L. pneumophila* were prepared at various time points after growth at 37°C. Briefly, bacterial cell pellets were resuspended in 1 ml of lysis buffer and sonicated for 2 min. The cells were then centrifuged for 1 h at 12,000× g. The protein-containing supernatant was removed and the protein concentration was measured using a commercial kit (Biorad Co.). Samples were normalized for protein loading and run on a 15% sodium dodecyl sulfate-polyacrylamide gel (SDS-PAGE) as described previously (Laemmli, 1970). Western blotting was carried out as described elsewhere (Towbin et al., 1979). The levels of CsrA or IHFB were immunoblotted with anti-His tag antibody. ICDH was probed as a loading control.

### Expression and Purification of IHFB

The coding regions of IHFB were PCR amplified and fused to the expression vector pET-28a by Gibson assembly method. The fusion gene constructs were transformed into *E. coli* strain BL21. For protein expression, 5 ml of overnight culture of the *E. coli* cells harboring the appropriate plasmid was transferred to 500 ml of LB medium with 50 μg/ml kanamycin and grown until OD_600_ of 0.4–0.6 was reached. After adding IPTG (isopropyl thio-d-galactopyranoside) to a final concentration of 10 μM, the cultures were further incubated in a shaker at 20°C for 16–18 h. Bacterial cells were harvested by spinning at 5000× g, resuspended in lysis buffer (25 mM Tris–HCl and 500 mM NaCl), and lysed by sonication. The soluble fractions were collected by centrifugation at 12,000× g for 30 min at 4°C. His-tagged proteins were purified with Ni-NTA affinity column (GE Healthcare Co.) following the manufacturer's instructions.

### Electrophoretic Mobility Shift Assay (EMSA)

EMSA was performed as previously described (Altman and Segal, 2008), with a few modifications. The His-tagged IHFB protein was purified with Ni-NTA affinity column (GE Healthcare) following the manufacturer's instructions. The regulatory region of *csrA* (~600 bp) was amplified by PCR with primers listed in Supplementary Table S2. Increasing amounts of the purified protein were mixed with 200 ng of the *csrA* promoter region probe in buffer containing 10 mM Tris–HCl (pH 7.5), 50 mM KCl, 5 mM MgCl_2_, 0.1 mM EDTA, 0.1 mM dithiothreitol, 0.1 mg/ml bovine serum albumin, 0.5 mg/ml herring sperm DNA, and 5% glycerol. The binding reaction was carried out for 30 min at room temperature, and samples were then loaded onto 6% polyacrylamide 0.5× Tris–acetate–EDTA gel in 0.5× Tris–acetate–EDTA running buffer. Following electrophoresis at 4°C, the gel was transferred to nylon membrane and fixed by UV cross-linking.

### GFP Reporter Assay

To confirm the regulation of *csrA* transcription by IHFB *via* a ClpP-dependent manner, a fragment containing 600 bp of the *csrA* RBS (Heuner et al., 1995), the putative σ70 promoter and transcriptional start site was amplified using primers P*PcsrA*-F and P*PcsrA*-R. The fragment was ligated into pJB908 directly 5′ of *gfp* as described (Hammer and Swanson, 1999). The plasmid pJB908-*PcsrA-gfp* was transformed into WT and Δ*clpP*, respectively, generating WT/p*PcsrA-gfp* and Δ*clpP*/p*PcsrA-gfp*.

### Statistical Analysis

Basic statistical analysis was performed using Excel. One-way ANOVA was performed using GraphPad Prism followed by a *post-hoc* Student–Newman–Keul's test. Morphological length of bacteria and quantitative analysis of Western blot were performed using ImageJ. The alignment of amino acid sequences was performed using the online NCBI BLAST.

## Data Availability Statement

The datasets generated for this study can be found in the LP02 (https://www.ncbi.nlm.nih.gov/sra/SRX5381953[accn]), XP02 (https://www.ncbi.nlm.nih.gov/sra/?term=PRJNA522681).

## Author Contributions

YL conceived the project. YL and ZG designed the experiments. ZG, QL, PY, XP, and DS performed the experiments. YL and ZG prepared the manuscript.

### Conflict of Interest

The authors declare that the research was conducted in the absence of any commercial or financial relationships that could be construed as a potential conflict of interest.

## References

[B1] Ali Azam T. Iwata A. Nishimura A. Ueda S. Ishihama A. (1999). Growth phase-dependent variation in protein composition of the *Escherichia coli* nucleoid. J. Bacteriol. 181, 6361–6370. 10515926 10.1128/jb.181.20.6361-6370.1999 PMC103771

[B2] Al-Khodor S. Price C. T. Habyarimana F. Kalia A. Abu Kwaik Y. (2008). A Dot/Icm-translocated ankyrin protein of *Legionella pneumophila* is required for intracellular proliferation within human macrophages and protozoa. Mol. Microbiol. 70, 908–923. 10.1111/j.1365-2958.2008.06453.x 18811729 PMC3064707

[B3] Altman E. Segal G. (2008). The response regulator CpxR directly regulates expression of several *Legionella pneumophilaicm*/*dot* components as well as new translocated substrates. J. Bacteriol. 190, 1985–1996. 10.1128/JB.01493-07 18192394 PMC2258895

[B4] Arvizu-Gomez J. L. Hernandez-Morales A. Pastor-Palacios G. Brieba L. G. Alvarez-Morales A. (2011). Integration host factor (IHF) binds to the promoter region of the *phtD* operon involved in phaseolotoxin synthesis in *P. syringae* pv. phaseolicola NPS3121. BMC Microbiol. 11:90. 10.1186/1471-2180-11-90 21542933 PMC3112066

[B5] Bachman M. A. Swanson M. S. (2004). The LetE protein enhances expression of multiple LetA/LetS-dependent transmission traits by *Legionella pneumophila*. Infect. Immun. 72, 3284–3293. 10.1128/IAI.72.6.3284-3293.2004 15155631 PMC415668

[B6] Bruggemann H. Hagman A. Jules M. Sismeiro O. Dillies M. A. Gouyette C.. (2006). Virulence strategies for infecting phagocytes deduced from the *in vivo* transcriptional program of *Legionella pneumophila*. Cell Microbiol. 8, 1228–1240. 10.1111/j.1462-5822.2006.00703.x 16882028

[B7] Byrne B. Swanson M. S. (1998). Expression of *Legionella pneumophila* virulence traits in response to growth conditions. Infect. Immun. 66, 3029–3034. 9632562 10.1128/iai.66.7.3029-3034.1998 PMC108309

[B8] Craig N. L. Nash H. A. (1984). *E. coli* integration host factor binds to specific sites in DNA. Cell 39, 707–716. 10.1016/0092-8674(84)90478-1 6096022

[B9] Dorman C. J. McKenna S. Beloin C. (2001). Regulation of virulence gene expression in *Shigella flexneri*, a facultative intracellular pathogen. Int. J. Med. Microbiol. 291, 89–96. 10.1078/1438-4221-00105 11437343

[B10] Faucher S. P. Mueller C. A. Shuman H. A. (2011). *Legionella pneumophila* transcriptome during intracellular multiplication in human macrophages. Front. Microbiol. 2:60. 10.3389/fmicb.2011.00060 21747786 PMC3128937

[B11] Feeley J. C. Gibson R. J. Gorman G. W. Langford N. C. Rasheed J. K. Mackel D. C.. (1979). Charcoal-yeast extract agar: primary isolation medium for *Legionella pneumophila*. J. Clin. Microbiol. 10, 437–441. 393713 10.1128/jcm.10.4.437-441.1979 PMC273193

[B12] Feng J. Michalik S. Varming A. N. Andersen J. H. Albrecht D. Jelsbak L.. (2013). Trapping and proteomic identification of cellular substrates of the ClpP protease in *Staphylococcus aureus*. J. Proteome Res. 12, 547–558. 10.1021/pr300394r 23253041

[B13] Fettes P. S. Forsbach-Birk V. Lynch D. Marre R. (2001). Overexpresssion of a *Legionella pneumophila* homologue of the *E. coli* regulator csrA affects cell size, flagellation, and pigmentation. Int. J. Med. Microbiol. 291, 353–360. 10.1078/1438-4221-00141 11727819

[B14] Fields B. S. Benson R. F. Besser R. E. (2002). *Legionella* and Legionnaires' disease: 25 years of investigation. Clin. Microbiol. Rev. 15, 506–526. 10.1128/CMR.15.3.506-526.2002 12097254 PMC118082

[B15] Flynn J. M. Neher S. B. Kim Y. I. Sauer R. T. Baker T. A. (2003). Proteomic discovery of cellular substrates of the ClpXP protease reveals five classes of ClpX-recognition signals. Mol. Cell. 11, 671–683. 10.1016/S1097-2765(03)00060-1 12667450

[B16] Forsbach-Birk V. McNealy T. Shi C. Lynch D. Marre R. (2004). Reduced expression of the global regulator protein CsrA in *Legionella pneumophila* affects virulence-associated regulators and growth in *Acanthamoeba castellanii*. Int. J. Med. Microbiol. 294, 15–25. 10.1016/j.ijmm.2003.12.003 15293450

[B17] Fyfe J. A. Davies J. K. (1998). An AT-rich tract containing an integration host factor-binding domain and two UP-like elements enhances transcription from the pilEp1 promoter of *Neisseria gonorrhoeae*. J. Bacteriol. 180, 2152–2159. 9555899 10.1128/jb.180.8.2152-2159.1998 PMC107143

[B18] Gal-Mor O. Segal G. (2003). Identification of CpxR as a positive regulator of *icm* and *dot* virulence genes of *Legionella pneumophila*. J. Bacteriol. 185, 4908–4919. 10.1128/JB.185.16.4908-4919.2003 12897011 PMC166489

[B19] Gober J. W. Shapiro L. (1990). Integration host factor is required for the activation of developmentally regulated genes in *Caulobacter*. Genes. Dev. 4, 1494–1504. 10.1101/gad.4.9.1494 2253876

[B20] Goodman S. D. Velten N. J. Gao Q. Robinson S. Segall A. M. (1999). *In vitro* selection of integration host factor binding sites. J. Bacteriol. 181, 3246–3255. 10322029 10.1128/jb.181.10.3246-3255.1999 PMC93783

[B21] Goosen N. van de Putte P. (1995). The regulation of transcription initiation by integration host factor. Mol. Microbiol. 16, 1–7. 10.1111/j.1365-2958.1995.tb02386.x 7651128

[B22] Gottesman S. (2003). Proteolysis in bacterial regulatory circuits. Annu. Rev. Cell Dev. Biol. 19, 565–587. 10.1146/annurev.cellbio.19.110701.153228 14570582

[B23] Guyard C. Low D. E. (2011). *Legionella* infections and travel associated legionellosis. Travel Med. Infect. Dis. 9, 176–186. 10.1016/j.tmaid.2010.05.006 21995862

[B24] Hales L. M. Shuman H. A. (1999). The *L. pneumophilarpoS* gene is required for growth within *Acanthamoeba castellanii*. J. Bacteriol. 181, 4879–4889. 10438758 10.1128/jb.181.16.4879-4889.1999 PMC93975

[B25] Hammer B. K. Swanson M. S. (1999). Co-ordination of *Legionella pneumophila* virulence with entry into stationary phase by ppGpp. Mol. Microbiol. 33, 721–731. 10.1046/j.1365-2958.1999.01519.x 10447882

[B26] Hengge R. (2009). Proteolysis of sigmaS (RpoS) and the general stress response in *Escherichia coli*. Res. Microbiol. 160, 667–676. 10.1016/j.resmic.2009.08.014 19765651

[B27] Heuner K. Bender-Beck L. Brand B. C. Luck P. C. Mann K. H. Marre R.. (1995). Cloning and genetic characterization of the flagellum subunit gene (*flaA*) of *Legionella pneumophila* serogroup 1. Infect. Immun. 63, 2499–2507. 7790062 10.1128/iai.63.7.2499-2507.1995 PMC173334

[B28] Jacobi S. Schade R. Heuner K. (2004). Characterization of the alternative sigma factor sigma54 and the transcriptional regulator FleQ of *Legionella pneumophila*, which are both involved in the regulation cascade of flagellar gene expression. J. Bacteriol. 186, 2540–2547. 10.1128/JB.186.9.2540-2547.2004 15090493 PMC387802

[B29] Joshi K. K. Chien P. (2016). Regulated proteolysis in bacteria: *Caulobacter*. Annu. Rev. Genet. 50, 423–445. 10.1146/annurev-genet-120215-035235 27893963 PMC5510660

[B30] Knudsen G. M. Nielsen M. B. Thomsen L. E. Aabo S. Rychlik I. Olsen J. E. (2014). The role of ClpP, RpoS and CsrA in growth and filament formation of *Salmonella enterica* serovar Typhimurium at low temperature. BMC Microbiol. 14:208. 10.1186/s12866-014-0208-4 25123657 PMC4236599

[B31] Knudsen G. M. Olsen J. E. Aabo S. Barrow P. Rychlik I. Thomsen L. E. (2013). ClpP deletion causes attenuation of *Salmonella* Typhimurium virulence through mis-regulation of RpoS and indirect control of CsrA and the SPI genes. Microbiology 159, 1497–1509. 10.1099/mic.0.065797-0 23676436

[B32] Laemmli U. K. (1970). Cleavage of structural proteins during the assembly of the head of bacteriophage T4. Nature 227, 680–685. 10.1038/227680a0 5432063

[B33] LeBlanc J. J. Brassinga A. K. Ewann F. Davidson R. J. Hoffman P. S. (2008). An ortholog of OxyR in *Legionella pneumophila* is expressed postexponentially and negatively regulates the alkyl hydroperoxide reductase (*ahpC2D*) operon. J. Bacteriol. 190, 3444–3455. 10.1128/JB.00141-08 18359810 PMC2394990

[B34] Li H. Durbin R. (2009). Fast and accurate short read alignment with Burrows-Wheeler transform. Bioinformatics 25, 1754–1760. 10.1093/bioinformatics/btp324 19451168 PMC2705234

[B35] Li X. H. Zeng Y. L. Gao Y. Zheng X. C. Zhang Q. F. Zhou S. N.. (2010). The ClpP protease homologue is required for the transmission traits and cell division of the pathogen *Legionella pneumophila*. BMC Microbiol. 10:54. 10.1186/1471-2180-10-54 20167127 PMC2838875

[B36] Mahmoud S. A. Chien P. (2018). Regulated proteolysis in bacteria. Annu. Rev. Biochem. 87, 677–696. 10.1146/annurev-biochem-062917-012848 29648875 PMC6013389

[B37] Mangan M. W. Lucchini S. Danino V. Croinin T. O. Hinton J. C. Dorman C. J. (2006). The integration host factor (IHF) integrates stationary-phase and virulence gene expression in *Salmonella enterica* serovar Typhimurium. Mol. Microbiol. 59, 1831–1847. 10.1111/j.1365-2958.2006.05062.x 16553887

[B38] McKenna A. Hanna M. Banks E. Sivachenko A. Cibulskis K. Kernytsky A.. (2010). The genome analysis toolkit: a MapReduce framework for analyzing next-generation DNA sequencing data. Genome Res. 20, 1297–1303. 10.1101/gr.107524.110 20644199 PMC2928508

[B39] Molofsky A. B. Swanson M. S. (2003). *Legionella pneumophila* CsrA is a pivotal repressor of transmission traits and activator of replication. Mol. Microbiol. 50, 445–461. 10.1046/j.1365-2958.2003.03706.x 14617170 PMC13227487

[B40] Molofsky A. B. Swanson M. S. (2004). Differentiate to thrive: lessons from the *Legionella pneumophila* life cycle. Mol. Microbiol. 53, 29–40. 10.1111/j.1365-2958.2004.04129.x 15225301 PMC13218203

[B41] Morash M. G. Brassinga A. K. Warthan M. Gourabathini P. Garduno R. A. Goodman S. D.. (2009). Reciprocal expression of integration host factor and HU in the developmental cycle and infectivity of *Legionella pneumophila*. Appl. Environ. Microbiol. 75, 1826–1837. 10.1128/AEM.02756-08 19201975 PMC2663186

[B42] Neher S. B. Villen J. Oakes E. C. Bakalarski C. E. Sauer R. T. Gygi S. P.. (2006). Proteomic profiling of ClpXP substrates after DNA damage reveals extensive instability within SOS regulon. Mol. Cell 22, 193–204. 10.1016/j.molcel.2006.03.007 16630889

[B43] Newton H. J. Ang D. K. van Driel I. R. Hartland E. L. (2010). Molecular pathogenesis of infections caused by *Legionella pneumophila*. Clin. Microbiol. Rev. 23, 274–298. 10.1128/CMR.00052-09 20375353 PMC2863363

[B44] Oliva G. Sahr T. Buchrieser C. (2018). The life cycle of *L. pneumophila*: cellular differentiation is linked to virulence and metabolism. Front. Cell Infect. Microbiol. 8:3. 10.3389/fcimb.2018.00003 29404281 PMC5780407

[B45] Perez-Rueda E. Janga S. C. Martinez-Antonio A. (2009). Scaling relationship in the gene content of transcriptional machinery in bacteria. Mol. Biosyst. 5, 1494–1501. 10.1039/b907384a 19763344

[B46] Pitre C. A. Tanner J. R. Patel P. Brassinga A. K. (2013). Regulatory control of temporally expressed integration host factor (IHF) in *Legionella pneumophila*. Microbiology 159, 475–492. 10.1099/mic.0.062117-0 23288541

[B47] Porankiewicz J. Schelin J. Clarke A. K. (1998). The ATP-dependent Clp protease is essential for acclimation to UV-B and low temperature in the *cyanobacterium Synechococcus*. Mol. Microbiol. 29, 275–283. 10.1046/j.1365-2958.1998.00928.x 9701820

[B48] Porter M. E. Dorman C. J. (1997). Positive regulation of *Shigella flexneri* virulence genes by integration host factor. J. Bacteriol. 179, 6537–6550. 10.1128/jb.179.21.6537-6550.1997 9352898 PMC179577

[B49] Quon K. C. Marczynski G. T. Shapiro L. (1996). Cell cycle control by an essential bacterial two-component signal transduction protein. Cell 84, 83–93. 10.1016/S0092-8674(00)80995-2 8548829

[B50] Rausch T. Zichner T. Schlattl A. Stutz A. M. Benes V. Korbel J. O. (2012). DELLY: structural variant discovery by integrated paired-end and split-read analysis. Bioinformatics 28, i333–i339. 10.1093/bioinformatics/bts378 22962449 PMC3436805

[B51] Robertson G. T. Ng W. L. Foley J. Gilmour R. Winkler M. E. (2002). Global transcriptional analysis of *clpP* mutations of type 2 *Streptococcus pneumoniae* and their effects on physiology and virulence. J. Bacteriol. 184, 3508–3520. 10.1128/JB.184.13.3508-3520.2002 12057945 PMC135132

[B52] Sahr T. Bruggemann H. Jules M. Lomma M. Albert-Weissenberger C. Cazalet C.. (2009). Two small ncRNAs jointly govern virulence and transmission in *Legionella pneumophila*. Mol. Microbiol. 72, 741–762. 10.1111/j.1365-2958.2009.06677.x 19400772 PMC2888818

[B53] Sahr T. Rusniok C. Impens F. Oliva G. Sismeiro O. Coppee J. Y.. (2017). The *Legionella pneumophila* genome evolved to accommodate multiple regulatory mechanisms controlled by the CsrA-system. PLoS Genet. 13:e1006629. 10.1371/journal.pgen.1006629 28212376 PMC5338858

[B54] Segal G. Shuman H. A. (1999). *Legionella pneumophila* utilizes the same genes to multiply within *Acanthamoeba castellanii* and human macrophages. Infect. Immun. 67, 2117–2124. 10225863 10.1128/iai.67.5.2117-2124.1999 PMC115946

[B55] Sexton J. A. Pinkner J. S. Roth R. Heuser J. E. Hultgren S. J. Vogel J. P. (2004). The *Legionella pneumophila* PilT homologue DotB exhibits ATPase activity that is critical for intracellular growth. J. Bacteriol. 186, 1658–1666. 10.1128/JB.186.6.1658-1666.2004 14996796 PMC355965

[B56] Siam R. Brassinga A. K. Marczynski G. T. (2003). A dual binding site for integration host factor and the response regulator CtrA inside the *Caulobacter crescentus* replication origin. J. Bacteriol. 185, 5563–5572. 10.1128/JB.185.18.5563-5572.2003 12949109 PMC193745

[B57] Sieira R. Comerci D. J. Pietrasanta L. I. Ugalde R. A. (2004). Integration host factor is involved in transcriptional regulation of the *Brucella abortusvirB* operon. Mol. Microbiol. 54, 808–822. 10.1111/j.1365-2958.2004.04316.x 15491369

[B58] Stonehouse E. Kovacikova G. Taylor R. K. Skorupski K. (2008). Integration host factor positively regulates virulence gene expression in *Vibrio cholerae*. J. Bacteriol. 190, 4736–4748. 10.1128/JB.00089-08 18456804 PMC2446820

[B59] Tan I. S. Weiss C. A. Popham D. L. Ramamurthi K. S. (2015). A quality-control mechanism removes unfit cells from a population of sporulating bacteria. Dev. Cell 34, 682–693. 10.1016/j.devcel.2015.08.009 26387458 PMC4588057

[B60] Tiaden A. Spirig T. Weber S. S. Bruggemann H. Bosshard R. Buchrieser C.. (2007). The *Legionella pneumophila* response regulator LqsR promotes host cell interactions as an element of the virulence regulatory network controlled by RpoS and LetA. Cell Microbiol. 9, 2903–2920. 10.1111/j.1462-5822.2007.01005.x 17614967

[B61] Towbin H. Staehelin T. Gordon J. (1979). Electrophoretic transfer of proteins from polyacrylamide gels to nitrocellulose sheets: procedure and some applications. Proc. Natl. Acad. Sci. U.S.A. 76, 4350–4354. 10.1073/pnas.76.9.4350 388439 PMC411572

[B62] Vakulskas C. A. Potts A. H. Babitzke P. Ahmer B. M. Romeo T. (2015). Regulation of bacterial virulence by Csr (Rsm) systems. Microbiol. Mol. Biol. Rev. 79, 193–224. 10.1128/MMBR.00052-14 25833324 PMC4394879

[B63] Yakhnin H. Yakhnin A. V. Baker C. S. Sineva E. Berezin I. Romeo T. (2011). Complex regulation of the global regulatory gene csrA: CsrA-mediated translational repression, transcription from five promoters by Eσ^70^ and Eσ^*S*^, and indirect transcriptional activation by CsrA. Mol. Microbiol. 81, 689–704. 10.1111/j.1365-2958.2011.07723.x 21696456 PMC3189700

[B64] Zhao B. B. Li X. H. Zeng Y. L. Lu Y. J. (2016). ClpP-deletion impairs the virulence of *Legionella pneumophila* and the optimal translocation of effector proteins. BMC Microbiol. 16:174. 10.1186/s12866-016-0790-8 27484084 PMC4969725

[B65] Zusman T. Aloni G. Halperin E. Kotzer H. Degtyar E. Feldman M.. (2007). The response regulator PmrA is a major regulator of the *icm*/*dot* type IV secretion system in*Legionella pneumophila* and *Coxiella burnetii*. Mol. Microbiol. 63, 1508–1523. 10.1111/j.1365-2958.2007.05604.x 17302824

