# Supplementary material for: The Temporal Expression of Global Regulator Protein CsrA Is Dually Regulated by ClpP During the Biphasic Life Cycle of Legionella pneumophila
Source: Front Microbiol. 2019 Nov 7;10:2495. doi: 10.3389/fmicb.2019.02495 (PMC6853998; doi:10.3389/fmicb.2019.02495)
Supplement: Supplementary file 1 [file Data_Sheet_1.PDF]

Supplementary Material

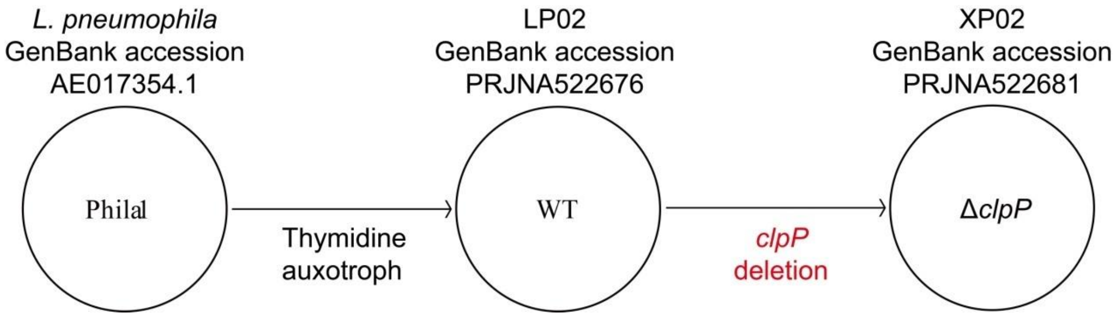

Supplementary Figure S1. Re-sequencing of the laboratory strains of *L. pneumophila* Philadelphia-1 reveals that only *clpP* gene was deleted in Δ*clpP* compared to WT.

The results of whole genome re-sequencing showed that the *clpP* is the only deleted gene in Δ*clpP* strain compared to WT strain, which not only supports the reliability of our previous reports (Li *et al.*, 2010, Zhao *et al.*, 2016), but also suggests that it is not necessary to include a *clpP* complement strain Δ*clpP*/C in the indicated experiments.
