# Supplementary material for: The Temporal Expression of Global Regulator Protein CsrA Is Dually Regulated by ClpP During the Biphasic Life Cycle of Legionella pneumophila
Source: Front Microbiol. 2019 Nov 7;10:2495. doi: 10.3389/fmicb.2019.02495 (PMC6853998; doi:10.3389/fmicb.2019.02495)
Supplement: Supplementary file 2 [file Data_Sheet_2.PDF]

Supplementary Material

A Representive peptide

```
1         11        21        31        41        51        61
MDIINLKFEE PLIIRISNTV VKILAFKTQE NGNIKFGVEA PRSINIHREE VFHAIKQKET LSTAD
```

B Δ*clpP*-TP

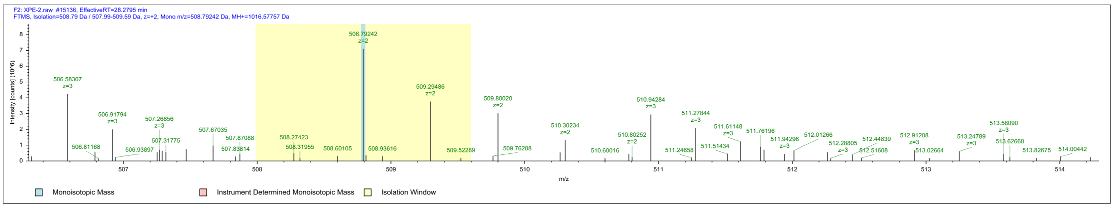

C WT-TP

No peptide

D WT-RP

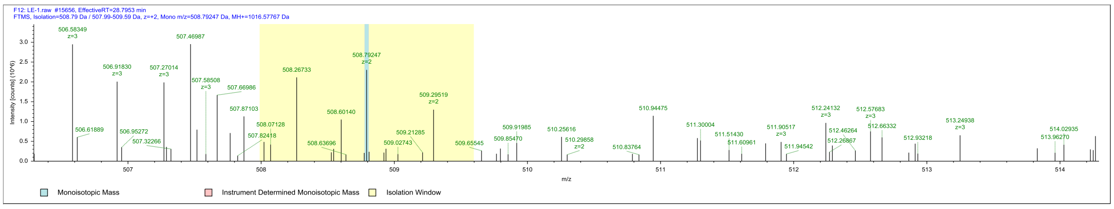

E Δ*clpP*-RP

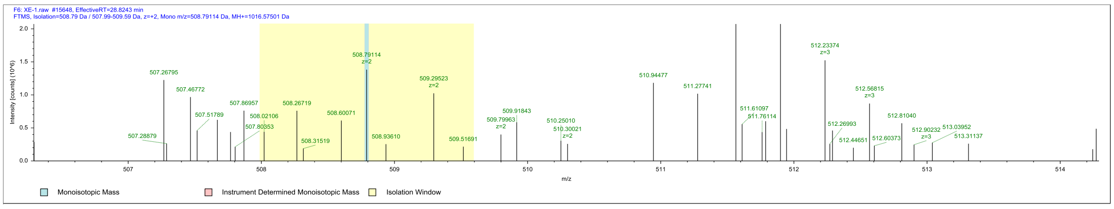

Supplementary Figure S2. Raw data of CsrA representive peptide by LC-MS in the RP and the TP of WT and Δ*clpP*.

(A). Representative peptides identified by LC-MS to CsrA are labeled green.

(B-E). Peptide fingerprint of CsrA in WT and Δ*clpP* at indicated growth phase. RP refers to the exponential growth of bacteria in AYE broth, and TP refers to the period approximately 6 h after the cessation of growth.
