# Supplementary material for: The Temporal Expression of Global Regulator Protein CsrA Is Dually Regulated by ClpP During the Biphasic Life Cycle of Legionella pneumophila
Source: Front Microbiol. 2019 Nov 7;10:2495. doi: 10.3389/fmicb.2019.02495 (PMC6853998; doi:10.3389/fmicb.2019.02495)
Supplement: Supplementary file 3 [file Data_Sheet_3.PDF]

Supplementary Material

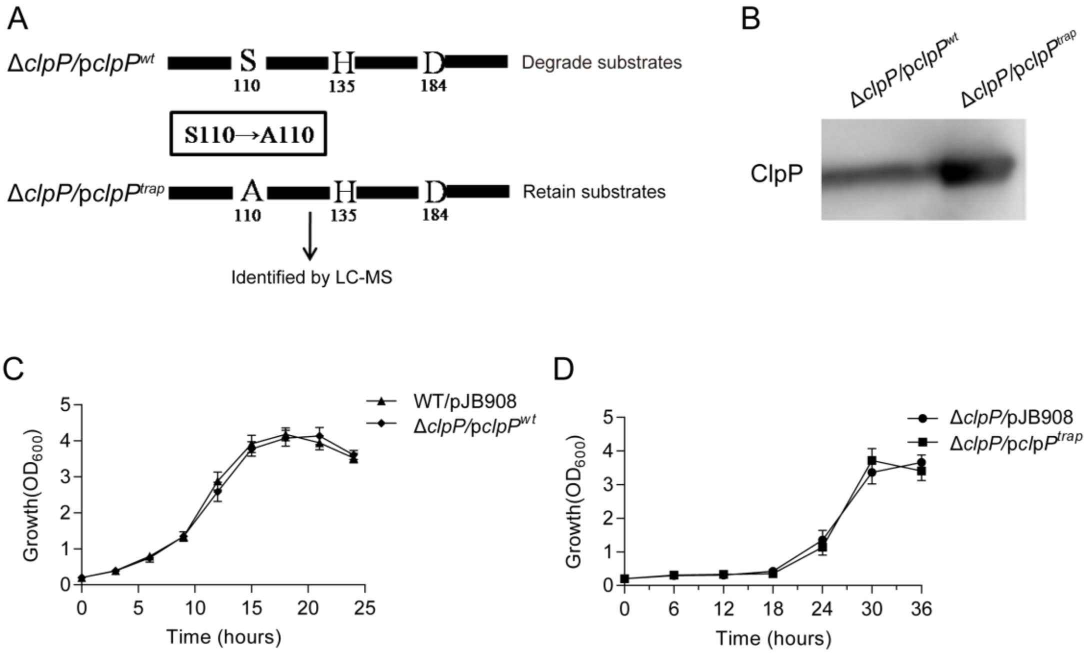

Supplementary Figure S3. Construction of the ClpP-Trap System successfully.

(A). Graphical representation of the biological construction of the ClpP-Trap system.

(B). Western blotting was used to detect the expression of ClpP in Δ*clpP*/p*clpP*^{wt} and Δ*clpP*/p*clpP*^{trap}.

(C). Growth curve measurement of WT/pJB908 (▲) and Δ*clpP*/p*clpP*^{wt} (◆).

(D). Growth curve measurement of Δ*clpP*/pJB908 (●) and Δ*clpP*/p*clpP*^{trap} (■).
