# Supplementary material for: The Temporal Expression of Global Regulator Protein CsrA Is Dually Regulated by ClpP During the Biphasic Life Cycle of Legionella pneumophila
Source: Front Microbiol. 2019 Nov 7;10:2495. doi: 10.3389/fmicb.2019.02495 (PMC6853998; doi:10.3389/fmicb.2019.02495)
Supplement: Supplementary file 5 [file Data_Sheet_5.PDF]

Supplementary Material

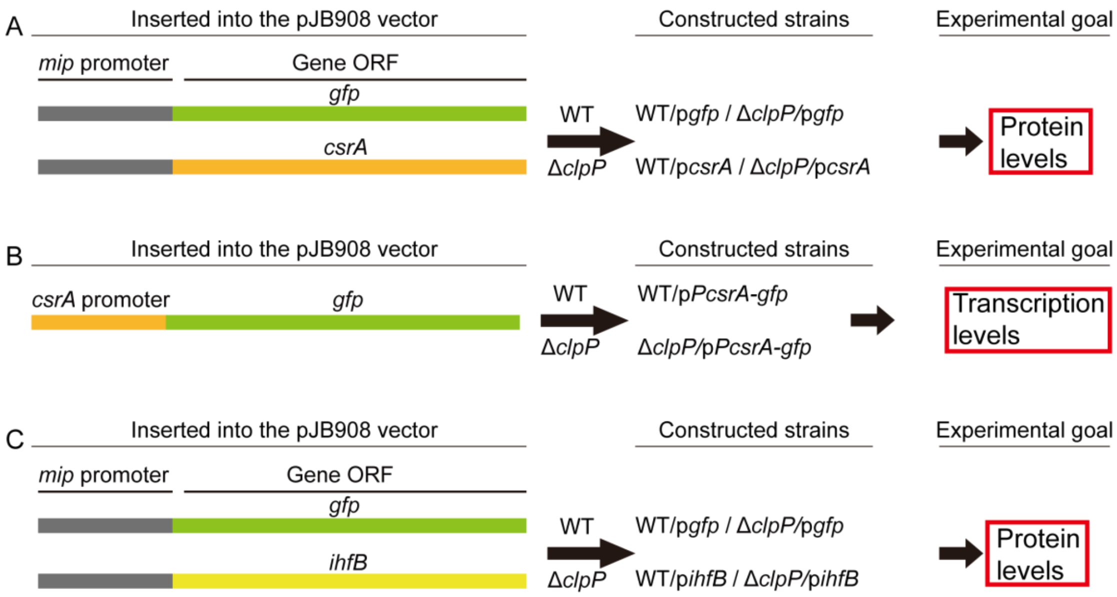

Supplementary Figure S5. Schematic of ClpP in regulating the expression of CsrA in both transcription and degradation

A. Under the control of the same promoter, the interference of transcription levels are excluded. Ectopic expression of CsrA and GFP, respectively, in WT and Δ*clpP*, detecting whether the protein level of expressed CsrA is regulated *via* ClpP-dependent manner.

B. Ectopic expression of GFP under the control of *csrA* promoter in WT and Δ*clpP*. GFP as a non-functional protein, the interference of the expressed CsrA on its own transcriptional regulation is excluded, further detecting that whether the transcription level of *csrA* is regulated *via* ClpP-dependent manner.

C. Under the control of the same promoter, the interference of transcription levels are excluded. Ectopic expression of IHFB and GFP, respectively, in WT and Δ*clpP*, detecting whether the protein level of expressed IHFB is regulated *via* ClpP-dependent manner.
