# Supplementary material for: The Temporal Expression of Global Regulator Protein CsrA Is Dually Regulated by ClpP During the Biphasic Life Cycle of Legionella pneumophila
Source: Front Microbiol. 2019 Nov 7;10:2495. doi: 10.3389/fmicb.2019.02495 (PMC6853998; doi:10.3389/fmicb.2019.02495)
Supplement: Supplementary file 6 [file Data_Sheet_6.PDF]

Supplementary Material

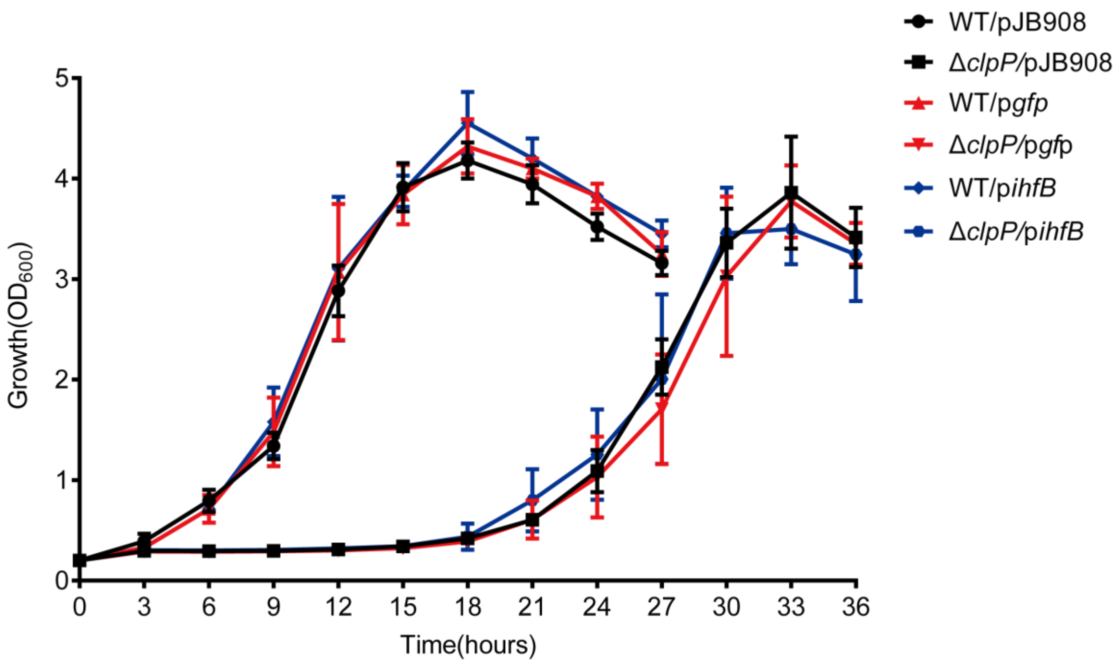

Supplementary Figure S6. The expression of GFP and IHFB does not affect the growth of *L. pneumophila* in both WT and Δ*clpP*, indicating that the affection of the bacterial growth by CsrA is regulated by ClpP, but not due to the stress caused by accumulation of overexpressed protein.

Growth curves of *L. pneumophila* wild-type strain WT (●), the *clpP* deletion mutant Δ*clpP* (■), WT with *gfp* expression (WT/p*gfp*) (▲), Δ*clpP* with *gfp* expression (Δ*clpP*/p*gfp*) (▼), WT with *ihfB* expression (WT/p*ihfB*) (◆) and Δ*clpP* with *ihfB* expression (Δ*clpP*/p*ihfB*) (■). For negative controls, pJB908 vector was electroporated into WT and Δ*clpP* to create WT/pJB908, Δ*clpP*/pJB908. Bacterial strains at TP (OD_{600}=3.0-3.5) were grown in AYE medium at 37 ℃ and samples were taken every 3 h for determination of optical density at 600 nm.
