# Supplementary material for: The Temporal Expression of Global Regulator Protein CsrA Is Dually Regulated by ClpP During the Biphasic Life Cycle of Legionella pneumophila
Source: Front Microbiol. 2019 Nov 7;10:2495. doi: 10.3389/fmicb.2019.02495 (PMC6853998; doi:10.3389/fmicb.2019.02495)
Supplement: Supplementary file 7 [file Data_Sheet_7.PDF]

Supplementary Material

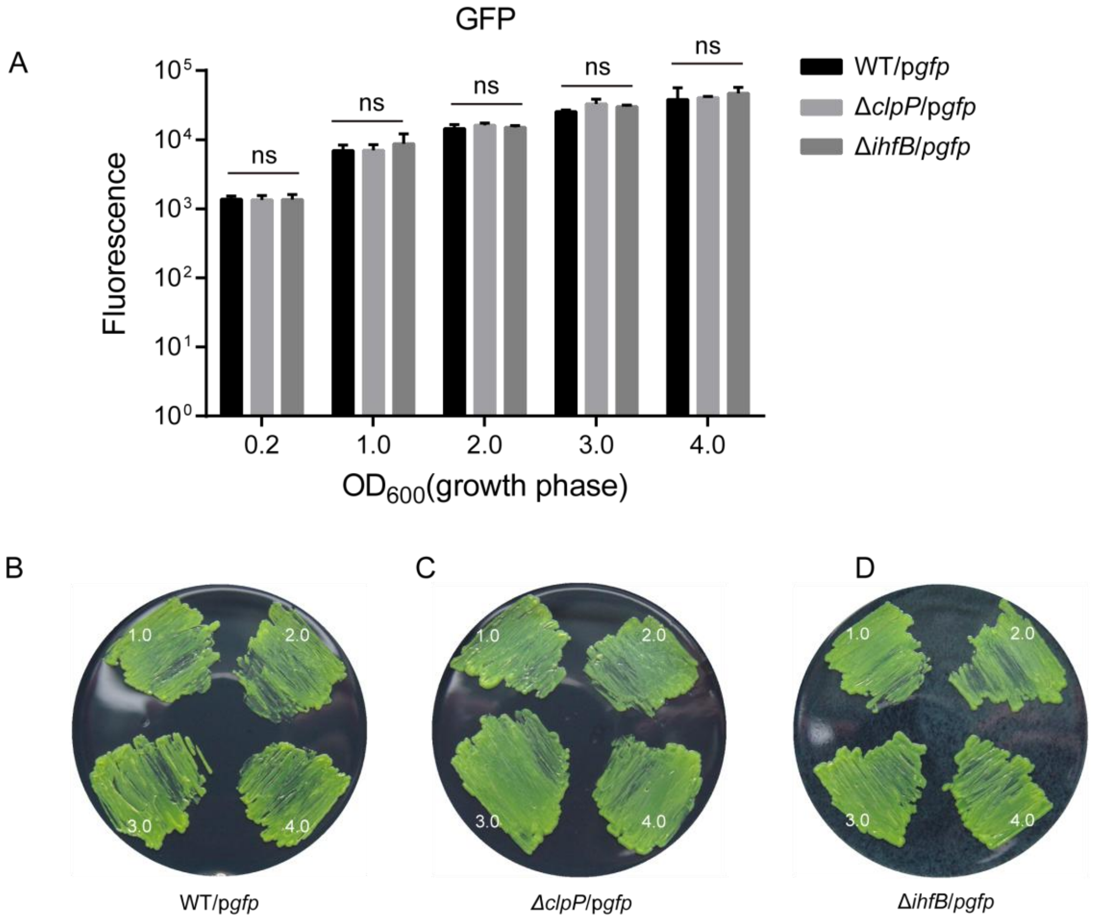

Supplementary Figure S7. The expression of GFP under the control of *mip* promoter in *L. pneumophila* shows no significant difference in the indicated strains at the same growth phase, demonstrating that either ClpP or IHFB do not affect the expression of *gfp* controlled by *mip* promoter at indicated growth phase

A. Detection of GFP expression in WT/p*gfp*, Δ*clpP*/p*gfp*, Δ*ihfB*/p*gfp* by fluorescence intensity analysis at the indicated OD_{600}. There has no significant difference in the expression levels of GFP in these strains at the same growth phase, indicating that the difference in expression of CsrA in WT, Δ*clpP* and Δ*ihfB* (Figure 1B and D; Figure 7) and the difference in expression of IHFB in WT and Δ*clpP* (Figure 6A and B) is regulated in a ClpP-dependent manner.

B-D. GFP expression of WT/p*gfp*, Δ*clpP*/p*gfp*, Δ*ihfB/pgfp* on BCYET plates. The numbers are the values of OD_{600}.
