# Supplementary material for: The Temporal Expression of Global Regulator Protein CsrA Is Dually Regulated by ClpP During the Biphasic Life Cycle of Legionella pneumophila
Source: Front Microbiol. 2019 Nov 7;10:2495. doi: 10.3389/fmicb.2019.02495 (PMC6853998; doi:10.3389/fmicb.2019.02495)
Supplement: Supplementary file 8 [file Data_Sheet_8.PDF]

Supplementary Material

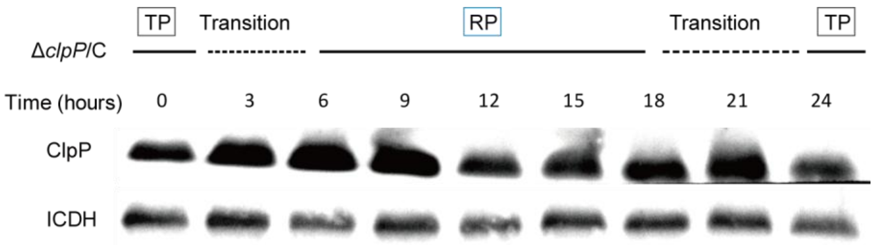

Supplementary Figure S8. The persistent expression of ClpP in Δ*clpP*/C during the life cycle demonstrates that ClpP plays a regulatory role throughout the life cycle

Bacterial whole-cell lysates at indicated time points were prepared from Δ*clpP*/C and an immunoblot of ClpP was performed using an anti-His tag antibody. ICDH was probed as a loading control. Each time point represents the mean plus standard deviation from three independent experiments.
