# Supplementary material for: The Temporal Expression of Global Regulator Protein CsrA Is Dually Regulated by ClpP During the Biphasic Life Cycle of Legionella pneumophila
Source: Front Microbiol. 2019 Nov 7;10:2495. doi: 10.3389/fmicb.2019.02495 (PMC6853998; doi:10.3389/fmicb.2019.02495)
Supplement: Supplementary file 9 [file Data_Sheet_9.PDF]

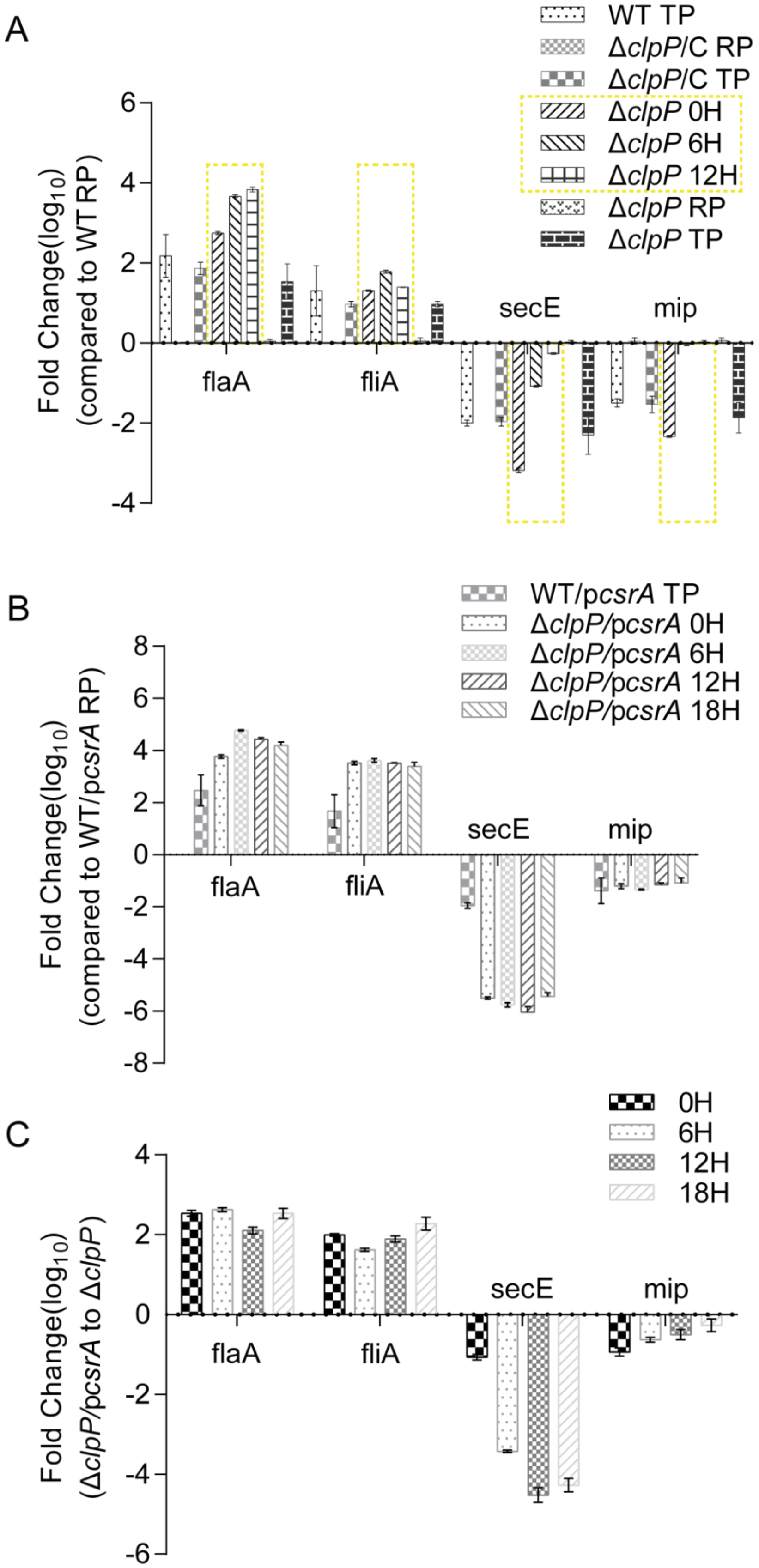

Supplementary Figure S9. The expression of phase-specific genes in indicated

strains and during various time points. These data, coupling with the results shown in Figure 3, indicate that *clpP* deletion (A) and CsrA of *clpP* deletion strains (B and C) prolongs the lag phase from transmissive phase to replicative phase during the life cycle, respectively

(A) Relative fold change of the TP-specific genes *flaA* and *fliA* and the RP-specific genes *mip* and *secE* of WT, Δ*clpP*/C, and Δ*clpP* at time points indicated were examined at the RP (OD_{600} of 0.7–1.0) and the TP (6 h after the cessation of growth) by qRT-PCR. Transcriptional levels of the genes of WT at the RP were normalized to 0 by taking log10.

(B) Relative fold change of expression of *flaA*, *fliA*, *mip* and *secE* of WT/p*csrA* and Δ*clpP*/p*csrA* at time points indicated were examined. Transcriptional levels of *csrA* of WT/p*csrA* at the RP were normalized to 0 by taking log10.

(C) Relative fold change of expression of *flaA*, *fliA*,*mip* and *secE* of Δ*clpP*/pJB908 and Δ*clpP*/p*csrA* were examined at time points indicated. Transcriptional levels of the genes of Δ*clpP*/pJB908 were respectively normalized to 0 by taking log10.
