# Supplementary material for: The Temporal Expression of Global Regulator Protein CsrA Is Dually Regulated by ClpP During the Biphasic Life Cycle of Legionella pneumophila
Source: Front Microbiol. 2019 Nov 7;10:2495. doi: 10.3389/fmicb.2019.02495 (PMC6853998; doi:10.3389/fmicb.2019.02495)
Supplement: Supplementary file 12 [file Data_Sheet_12.PDF]

Supplementary Material

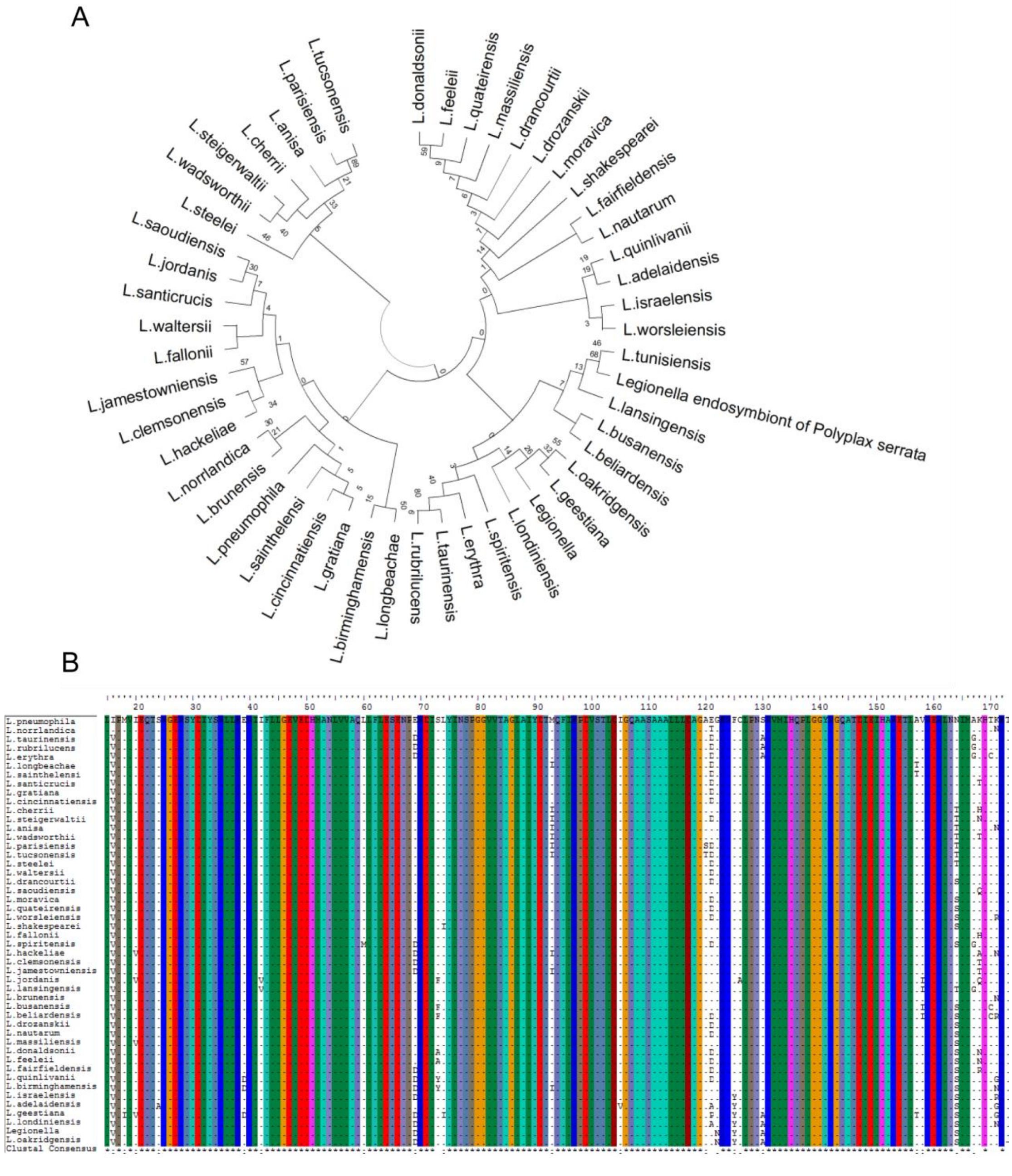

Supplementary Figure S12. ClpP is highly conserved in *Legionella* species.

(A) The evolutionary history was inferred using the Neighbor-Joining method. The optimal tree with the sum of branch length = 2.13048176 is shown. The percentage of replicate trees in which the associated tax a clustered together in the bootstrap test (2000 replicates) are shown next to the branches. The tree is drawn to scale, with branch lengths in the same units as those of the evolutionary distances used to infer the phylogenetic tree. The evolutionary distances were computed using the Poisson correction method and were in the units of the number of amino acid substitutions per site. This analysis involved 50 amino acid sequences. All positions containing gaps

and missing data were eliminated (complete deletion option). There was a total of 129 positions in the final dataset. Evolutionary analyses were conducted in MEGAX.

(B) Alignment of the amino acid sequence of ClpP in *Legionella* genera were conducted in BioEdit, and 48 genera were found to be highly conservative except for *L. tunisiensis* and *L. endosymbiont* of Polyplax serrata.
