# Supplementary material for: The Temporal Expression of Global Regulator Protein CsrA Is Dually Regulated by ClpP During the Biphasic Life Cycle of Legionella pneumophila
Source: Front Microbiol. 2019 Nov 7;10:2495. doi: 10.3389/fmicb.2019.02495 (PMC6853998; doi:10.3389/fmicb.2019.02495)
Supplement: Supplementary file 13 [file Data_Sheet_13.PDF]

Supplementary Material

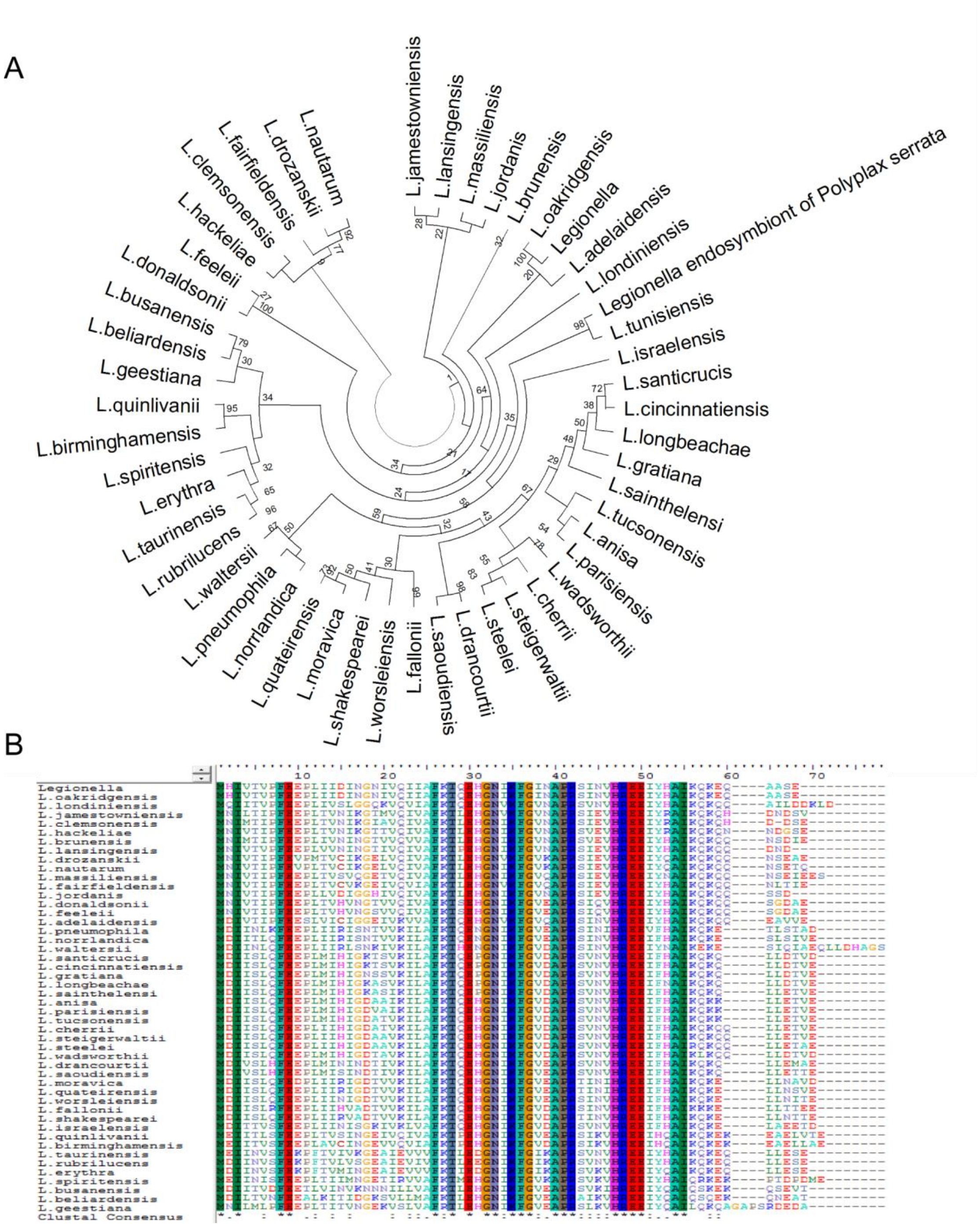

Supplementary Figure S13. CsrA is highly conserved in *Legionella* species.
(A) The evolutionary history was inferred using the Neighbor-Joining method. The bootstrap consensus tree inferred from 2000 replicates is taken to represent the evolutionary history of the taxa analyzed. Branches corresponding to partitions reproduced in less than 50% bootstrap replicates are collapsed. The percentage of replicate trees in which the associated taxa clustered together in the bootstrap test

(2000 replicates) are shown next to the branches. The evolutionary distances were computed using the Poisson correction method and are in the units of the number of amino acid substitutions per site. This analysis involved 50 amino acid sequences. All positions containing gaps and missing data were eliminated (complete deletion option). There was a total of 55 positions in the final dataset. Evolutionary analyses were conducted in MEGAX.

(B) Alignment of the amino acid sequence of CsrA in *Legionella* genera were conducted in BioEdit, and 48 genera were found to be conservative except for *L. tunisiensis* and *L. endosymbiont* of Polyplax serrata.
