# Supplementary material for: The Temporal Expression of Global Regulator Protein CsrA Is Dually Regulated by ClpP During the Biphasic Life Cycle of Legionella pneumophila
Source: Front Microbiol. 2019 Nov 7;10:2495. doi: 10.3389/fmicb.2019.02495 (PMC6853998; doi:10.3389/fmicb.2019.02495)
Supplement: Supplementary file 14 [file Table_1.DOCX]

***Supplementary Material***

**Supplementary Table S1. Bacterial strains and plasmids used in the study**

| **Strains or plasmids** | **Genotype or feature(s)^a^** | **Reference or source** | |
| --- | --- | --- | --- |
| **Strains**  WT  Δ*clpP*  Δ*clpP*/C  WT/pJB908  Δ*clpP*/pJB908  Δ*clpP*/p*clpP^wt^*  Δ*clpP*/p*clpP^trap^*  WT/p*csrA*  Δ*clpP*/p*csrA*  WT/p*ihfB*  Δ*clpP*/p*ihfB*  WT/p*gfp*  Δ*clpP*/p*gfp*  WT/p*PcsrA-gfp* | *L. pneumophila* serogroup 1, strain Philadelphia, *rpsL*, *HsdR^−^*, *Thy^−^* (LP02)  WT with *clpP* deletion (XP02)  Δ*clpP* containing *clpP* for complementation (XP02C)  WT containing pJB908 vector  Δ*clpP* containing pJB908 vector  Δ*clpP* with expression of *clpP* wild type gene with His-tag  Δ*clpP* with expression of *clpP* mutation gene (*S110→A110*) with His-tag  WT containing plasmids pJB908-*csrA* with expression of CsrA  Δ*clpP* containing plasmids pJB908-*csrA* with expression of CsrA  WT containing plasmids pJB908-*ihfB* with expression of IHFB  Δ*clpP* containing plasmids pJB908-*ihfB* with expression of IHFB  WT containing plasmids pJB908- *gfp* with expression of *gfp*  Δ*clpP* containing plasmids pJB908-*gfp* with expression of *gfp*  WT containing plasmids pJB908-*PcsrA-gfp* with expression of *gfp* under *csrA* promoter region | | Lab collection  Zhao *et al*. (2016)  Zhao *et al*. (2016)  This study  This study  This study  This study  This study  This study  This study  This study  This study  This study  This study |
| Δ*clpP*/p*PcsrA-gfp* | Δ*clpP* containing plasmids pJB908-*PcsrA-gfp* with expression of *gfp* under *csrA* promoter region | | This study |
| Δ*ihfB* | WT with *ihfB* deletion | | This study |
| Δ*ihfB/*pJB908 | Δ*ihfB* containing pJB908 vector | | This study |
| Δ*ihfB/*C | Δ*ihfB* containing plasmids pJB908-*ihfB/C* for complementation | | This study |
| Δ*ihfB/*p*csrA*  Δ*ihfB/*p*gfp* | Δ*ihfB* containing plasmids pJB908-*csrA* with expression of CsrA  Δ*ihfB* containing plasmids pJB908-*Pmip-gfp* with expression of *gfp* | | This study  This study |
| DH5α | F^-^ *endA1 hsdRI7* (r_k_^-^ m_k_^+^) *supE44 thi-1*λ^-^ *recA1 gyrA96* (Nal^r^) *relA1 Δ*(*lacZYA-argF*)*U169 deoR* φ80d*lacZ Δ*M15 | | Lab collection |
| BL21 | B F^-^ *ompT* *hsdS*(r_B_^-^ m_B_^-^) *dcm^+^* Tet^r^ *gal* λ(DE3) *endA* Hte [*argU ileY leuW* Cm^r^] | | Lab collection |
| BL21p*ihfB*-28a | BL21 containing plasmids pET-28a-*ihfB* with *in vitro* expression of IHFB | | This study |
| **Plasmids** |  | |  |
| pBRDX | Suicide delivery vector, *rdxA* *sacB* Cm | | LeBlanc *et al*. (2006) |
| pJB908 | Insert thy gene, mutate mob into pkB5 | | Sexton *et al*. (2004) |
| pJB908-*clpP^wt^*  pJB908-*clpP^trap^*  pJB908-*csrA* | pJB908 containing *clpP* (WT) with C-terminally His-tag  pJB908 containing *clpP* *(*mutant *, S110→A110)* with C-terminally His-tag  pJB908 containing *csrA* with C-terminally His-tag under the control of *mip* promoter | | This study  This study  This study |
| pJB908-*ihfB*  pJB908-*gfp* | pJB908 containing *ihfB* with C-terminally His-tag under the control of *mip* promoter  pJB908 containing *gfp* under the control of *mip* promoter | | This study  This study |
| pJB908-*PcsrA-gfp* | pJB908 containing *gfp* under the control of *csrA* promoter | | This study |
| pBRDXΔ*ihfB* | pBRDX::*clpP* for *clpP* deletion | | This study |
| pJB908-*ihfB/C* | Complementation plasmid, containing *ihfB* with its own promoter region | | This study |
| pET-28a-c(+) | Production of N-terminally His-tagged fusion proteins, P_T7_, Kan^r^ | | Novagen |
| pET-28a-*ihfB* | *In vitro* expression of *ihfB* with His-tag for EMSA | | This study |

^a^ Amp^r^, ampicillin resistant; Kan^r^ , kanamycin resistant; Cm^r^, chloramphenicol resistant; sacB^s^, sucrose sensitive.
