# Supplementary material for: The Temporal Expression of Global Regulator Protein CsrA Is Dually Regulated by ClpP During the Biphasic Life Cycle of Legionella pneumophila
Source: Front Microbiol. 2019 Nov 7;10:2495. doi: 10.3389/fmicb.2019.02495 (PMC6853998; doi:10.3389/fmicb.2019.02495)
Supplement: Supplementary file 15 [file Table_2.DOCX]

***Supplementary Material***

**Supplementary Table S2. Primers used in this study**

| **Primer** | **Primer sequence (5’→3’)** | **Purpose** | **Reference** |
| --- | --- | --- | --- |
| P*pmip*-F | TGGTGGGAGCTCGAAAAGGAATTTCTTTCTTGC | Insert *mip* promoter into pJB908 | This study |
| P*pmip*-R1 | TTAAGATTTATTATATCCATATGTATATCTCCTTCTTAAATCCCCTTTTAGTCTTACACT | Insert *mip* promoter into pJB908 | This study |
| P*clpP^wt^*-F  P*clpP^wt^*-R  P*clpP^trap^*-F  P*clpP^trap^*-R  P*csrA*-F | AGTGTAAGACTAAAAGGGGATTTAAGAAGGAGATATACATATGCCAGGCTATTCAGATAA  TGGTGGGCATGCTTAGTGATGATGATGATGATGACTACCACGTGGTACTAGTCCACTACTTAAGTCTGTAGAATGTCCAGC  GCTGCACGTGCTGCCTGGCCTATGCATAATGTACTTACAT  ATGTAAGTACATTATGCATAGGCCAGGCAGCACGTGCAGC  AGTGTAAGACTAAAAGGGGATTTAAGAAGGAGATATACATATGGATATAATAAATCTTAA | Expression of *clpP^wt^* with His-tag  Expression of *clpP^wt^* with His-tag  Expression of *clpP^trap^* with His-tag  Expression of *clpP^trap^* with His-tag  Expression of *csrA* with His-tag | This study  This study  This study  This study  This study |
| P*csrA*-R | TGGTGGGCATGCTTAGTGATGATGATGATGATGACTACCACGTGGTACTAGTCCACTACTGTCAGCTGTGCTAAGAGTTT | Expression of *csrA* with His-tag | This study |
| P*pmip*-R2 | ATGAGTTCGGATTTAATCATATGTATATCTCCTTCTTAAATCCCCTTTTAGTCTTACACT | Insert *mip* promoter into pJB908 | This study |
| P*ihfB*-F | AGTGTAAGACTAAAAGGGGATTTAAGAAGGAGATATACATATGATTAAATCCGAACTCAT | Expression of *ihfB* with His-tag | This study |
| P*ihfB*-R  P*pmip*-R3  Pm*gfp*-F  Pm*gfp*-R | GCTCTAGATTAGTGATGATGATGATGATGACTACCACGTGGTACTAGTCCACTACTATCTTTATCCAAAAGTGGGA  TCCTCGCCCTTGCTCACCATATGTATATCTCCTTCTTAAATCCCCTTTTAGTCTTACACT  AGTGTAAGACTAAAAGGGGATTTAAGAAGGAGATATACATATGGTGAGCAAGGGCGAGGA  GCTCTAGA TTACTTGTACAGCTCGTCCA | Expression of *ihfB* with His-tag  Insert *mip* promoter into pJB908  Expression of *gfp* under *Pmip*  Expression of *gfp* under *Pmip* | This study  This study  This study  This study |
| P*PcsrA*-F | TGGTGGGAGCTCTACGTTCATTAAGATATAATG | Insert *csrA* promoter into pJB908 | This study |
| P*PcsrA*-R | TCCTCGCCCTTGCTCACCATATGTATATCTCCTTCTTAAATTAAAAAAAACCTCTTTTTT | Insert *csrA* promoter into pJB908 | This study |
| P*gfp*-F | AAAAAAGAGGTTTTTTTTAATTTAAGAAGGAGATATACATATGGTGAGCAAGGGCGAGGA | Expression of *gfp* under *PcsrA* | This study |
| P*gfp*-R | GCTCTAGA TTACTTGTACAGCTCGTCCA | Expression of *gfp* under *PcsrA* | This study |
| PΔ*ihfB*-F1 | GAAGATCTGTAGGTATCGAATTGTTCCC | Deletion of the *ihfB* gene | This study |
| PΔ*ihfB*-R1 | CTTAGGATGAAAAATAAAATATACTCACCTCGTTTTGTCG | Deletion of the *ihfB* gene | This study |
| PΔ*ihfB*-F2 | CGACAAAACGAGGTGAGTATATTTTATTTTTCATCCTAAG | Deletion of the *ihfB* gene | This study |
| PΔ*ihfB*-R2 | CGGGATCCTAAGAAAAGAGGAATATCGT | Deletion of the *ihfB* gene | This study |
| P*ihfB*-CF | ggtaccgatctagatctcgagGTAGGTATCGAATTGTTCCC | Complementation of *ihfB* | This study |
| P*ihfB*-CR | gtgatgggttaaaaaggatccTAAGAAAAGAGGAATATCGT | Complementation of *ihfB* | This study |
| P*csrA* probe-F | TAGTAATCGCTTGTTTTTGAACA | Regulatory region of *csrA* | This study |
| P*csrA* probe-F | TTAAAAAAAACCTCTTTTTTCATAA | Regulatory region of *csrA* | This study |
| P*csrA-del* probe-F | AGGCAAGGGTTTTAAAAAGCTTATTAATGCTGCGTATCTT | Delete 60bp upstream from the transcriptional start site of *csrA* | This study |
| P*csrA-del* probe-R | AAGATACGCAGCATTAATAAGCTTTTTAAAACCCTTGCCT | Delete 60bp upstream from the transcriptional start site of *csrA* | This study |
| P*ihfB*-28a-F | CGCGGATCCATGATTAAATCCGAACTCAT | *In vitro* expression of *ihfB* | This study |
| P*ihfB*-28a-R | CCCAAGCTTATCTTTATCCAAAAGTGGGA | *In vitro* expression of *ihfB* | This study |
| *gfp*-qPCR-F | CAAGCAGAAGAACGGCATCAAG | qPCR analysis of the *gfp* mRNA | This study |
| *gfp*-qPCR-R | GATGGGGGTGTTCTGCTGGTAG | qPCR analysis of the *gfp* mRNA | This study |
| *csrA*-qPCR-F | GAACCACTTATCATTCGTATCTCG | qPCR analysis of the *csrA* mRNA | This study |
| *csrA*-qPCR-R | TTGATTGACCTGGGTGCCTC | qPCR analysis of the *csrA* mRNA | This study |
| *fliA*-qPCR-F | CACATCATCTGCTCGGGCG | qPCR analysis of the *fliA* mRNA | This study |
| *fliA*-qPCR-R | CATAAGTTTCAAAGGATGCCCCC | qPCR analysis of the *fliA* mRNA | This study |
| *flaA*-qPCR-F | TCAGTATCGGCAGCACAAAAGC | qPCR analysis of the *flaA* mRNA | This study |
| *flaA*-qPCR-R | CTTGTCCGTTTAGTGCCCCG | qPCR analysis of the *flaA* mRNA | This study |
| *mip*-qPCR-F | AGCATTGGTGCCGATTTGG | qPCR analysis of the *mip* mRNA | This study |
| *mip*-qPCR-R | CGCCACTCATAGCGTCTTGC | qPCR analysis of the *mip* mRNA | This study |
| *secE*-qPCR-F | GCCTACACGGCAAGAAACAATACA | qPCR analysis of the *secE* mRNA | This study |
| *secE*-qPCR-R | ATCCTGTCAATCCAACCATCACC | qPCR analysis of the *secE* mRNA | This study |
| 16S-qPCR-F | GAAGGGGCGACCTGGAGCAAAT | Determination of 16S half-life | This study |
| 16S-qPCR-R | ACACCGTGGTAAACGTCCCCCCGA | Determination of 16S half-life | This study |
