# Supplementary material for: The Temporal Expression of Global Regulator Protein CsrA Is Dually Regulated by ClpP During the Biphasic Life Cycle of Legionella pneumophila
Source: Front Microbiol. 2019 Nov 7;10:2495. doi: 10.3389/fmicb.2019.02495 (PMC6853998; doi:10.3389/fmicb.2019.02495)
Supplement: Supplementary file 11 [file Data_Sheet_11.PDF]

Supplementary Material

A Representive peptide

```
1          11         21         31         41         51         61
MIKSELIEHI AARMTHLTEK QVADGINRIL ELMSEALIHG QRIEIRGFGS FSLHYRPPRN AHNPKTGEKV
71         81         91
VTEAKYSPHF KPGKELRERV NSSRAKFPLLDKD
```

B Δ*clpP*/p*clpP*^{trap}

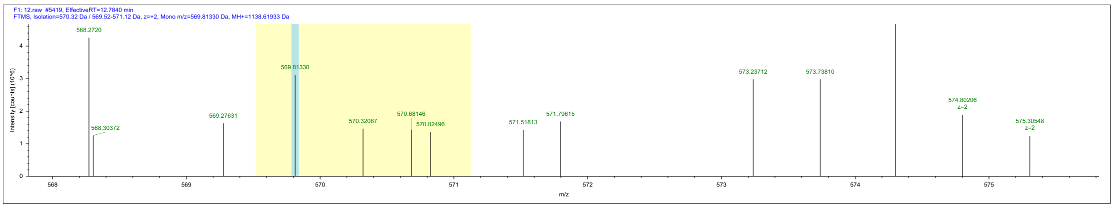

C Δ*clpP*/p*clpP*^{wt}

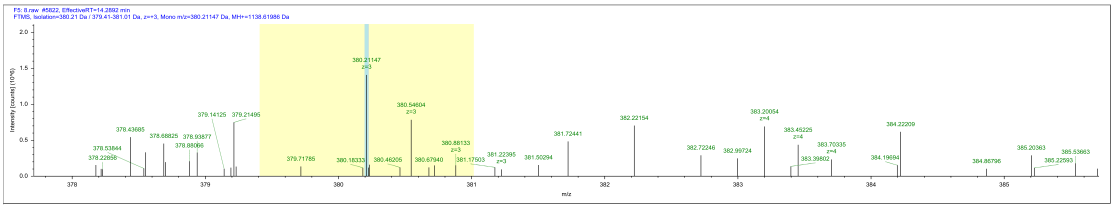

Supplementary Figure S11. Raw data of IHFB representive peptide by LC-MS in the TP of Δ*clpP*/p*clpP*^{trap} and Δ*clpP*/p*clpP*^{wt}.

(A). Representative peptides identified by LC-MS to IHFB are labeled green. The IHFB captured inside the proteolytic barrel were co-purified along with the His-tagged ClpP complex and identified by mass spectrometry.

(B-C). Peptide fingerprint of IHFB in Δ*clpP*/p*clpP*^{trap} (B) and Δ*clpP*/p*clpP*^{wt} (C).
